# Technological rejection in regions of early gold innovation revealed by geospatial analysis

**DOI:** 10.1038/s41598-021-98514-7

**Published:** 2021-10-13

**Authors:** Nathaniel L. Erb-Satullo

**Affiliations:** Cranfield Forensic Institute, Cranfield University, College Road, Cranfield, MK43 0AL UK

**Keywords:** Anthropology, Archaeology

## Abstract

In research on early invention and innovation, technological “firsts” receive enormous attention, but technological “lasts”—instances of abandonment and rejection—are arguably more informative about human technological behavior. Yet, cases of technological discontinuance are largely ignored in studies of early innovation, as the lack of robust datasets makes identification and analysis difficult. A large-scale geospatial analysis of more than 4500 gold objects from the Caucasus, an early center of gold innovation, shows a precipitous decline at 1500 BC in precisely the places with the earliest global evidence of gold mining (c. 3000 BC). Testing various causal models reveals that social factors, rather than resource limitations or demographic disruption, were the primary causes of this rejection. These results indicate that prior models of technological rejection and loss have underestimated the range of conditions in which they can occur, and provide empirical support for theories of innovation that reject notions about the linearity of technological progress.

## Introduction

The study of technological innovation—how inventions are generated and why they spread—is a major concern both within archaeology and in the broader social sciences. Early appearances of major innovations, from plant and animal domestication to material technologies such as pottery and metallurgy, have attracted intense interest^1–4^, but the last decade has seen renewed interest in invention and innovation as extended social and behavioral processes^5–9^. This trend in archaeological research has drawn on a variety of interdisciplinary research traditions from across the social and behavioral sciences^10–13^. The long-term perspective provided by archaeological data has enormous potential for the field of innovation research, which has largely focused on recent, successful, and rapidly spreading innovations. One common observation from the anthropology of technology is that technological development does not always follow predictable linear sequences from simple to complex, and the spread of innovations is never solely determined by objective measurements of efficiency divorced from social context^14,15^. Technologies can be discontinued, lost, abandoned, and rejected.

Despite widespread interest in innovation and the theoretical acknowledgement of non-linearity in technological change, systematic analysis of technological loss, abandonment, and rejection—particularly cases that occur after a period of sustained adoption—are rare^16,17^. Anecdotal observations of potential cases of technological loss are more common, as is research on cases of technological resistance and rejection of technologies among populations prior to adoption^18,19^. Yet, cases of technological discontinuance—the breakdown of technological systems—are highly informative about the mechanisms and dynamics of technological change. Research on the loss or rejection of innovations provides concrete data to counter popular notions of technology as a linear sequence of increasingly complex technologies, one leading seamlessly to another.

Systematic, empirical analysis of technological discontinuance is hindered by the patchiness of the archaeological record, complicating its identification and investigation. Without robust archaeological datasets, models remain largely untested theory, and may not accurately capture the range of conditions in which such discontinuities occur. Archaeological work not only expands the scope of innovation research beyond societies with substantial written accounts, it also helps to rectify the long-acknowledged pro-innovation bias in research on the diffusion of innovations^10,20^. Recently, spatial approaches to large datasets have proven useful for testing models of social and technological change using archaeological data^21–23^, highlighting the potential of these approaches for investigating technological loss.

The case of Caucasus goldwork presents an ideal opportunity to explore how and why technologies are lost or rejected after adoption. Almost from their first appearance in the archaeological record, gold artifacts were crucial elements of status, wealth, ritual, and prestige in many early complex societies worldwide^24–26^. Early gold mining and manufacturing required significant expertise, involved complex techniques, and entailed considerable effort^27^, linking gold production to issues of labor organization, technological innovation, and social hierarchy. The Caucasus was one of the first places in the world to use gold, with objects dating to the early 4th millennium BC^28^, only slightly later than the mid-5th millennium BC gold in the eastern Balkans, just across the Black Sea^24^. The Caucasus is also the location of the earliest known gold mine, dating to c. 3000 BC^27^. In later periods, the region’s gold was mythologized in the Greek myth of Jason, the Argonauts, and the Golden Fleece. Archaeological research has emphatically reinforced these legends, yielding a spectacular array of Bronze and Iron Age gold. In particular, the millennium between c. 2500 and 1500 BC witnessed an efflorescence of highly skilled gold-working, involving elaborate manufacturing technologies.

Yet, for at least 700 years after 1500 BC, some of these very same areas witnessed a precipitous decline in both the quantity and complexity of gold metallurgy. Paradoxically, Jason’s voyage, according to the mythical chronology, falls at a time of limited gold use in the supposed location of the Golden Fleece. Even more striking, this decline in gold-working diverges strongly from trajectories of copper-alloy metallurgy, which experienced a major expansion at this time^29^. On a superficial level, this paradox is resolved by pointing out that the Golden Fleece legend, or at least, the parts of it that identify the western Caucasus as its location, must have matured during the mid-1st millennium BC resurgence in gold metallurgy^30^. Nevertheless, major questions remain: how widespread was this discontinuity, what were its driving factors, and could it merely be an artifact of archaeological visibility and research patterns?

This study presents a comprehensive geospatial analysis of more than 4500 Bronze Age and Iron Age gold objects from the South Caucasus (modern-day Georgia, Armenia, and Azerbaijan). It explores the chronological and spatial parameters of this technological discontinuity in both quantitative and qualitative terms. Three explanatory models are tested against the data: a restriction of access to gold supplies, demographic factors resulting in a breakdown in technological transmission, and active rejection linked with mid-2nd millennium BC social transformations.

The data strongly suggest that the gold decline in the Middle Kura zone is *not* an artificial pattern related to archaeological visibility or ancient depositional practices, but rather a genuine case of technological rejection. Spatial modeling of access to gold deposits and analysis of archaeological data contradict the resource restriction and demographic models, and support a model of technological rejection in which social factors—particularly the involvement of goldwork in specific cultural practices emphasizing extreme social hierarchy—played a dominant role.

The discontinuance of gold metallurgy in parts of the South Caucasus is significant because it occurs in a period of demographic expansion, apparent prosperity, and considerable innovation in other metallurgical technologies. Thus, it differs from other models for social collapse and technological loss, suggesting that the range of conditions under which technological discontinuance can occur is underrecognized.

### Models of technological discontinuance

Technological discontinuance is a useful general term to refer to the cessation of a technological tradition, without implying the extent of intentionality (active rejection vs. passive loss) or introducing value-laden connotations (e.g. “de-innovation”). The terms discontinuance and discontinuity also carry an implicit critique of progressive, linear, unidirectional notions about technological change. The term implies a preceding period of at least partial adoption, which distinguishes it from the related, but distinct phenomena of non-adoption or delayed adoption. In contrast to technological discontinuance, non-adoption and delayed adoption—resistance to an innovation during the initial encounter—are better documented archaeologically^5,18,31,32^.

One cause of technological discontinuance, particularly in cases where the social desirability of and demand for a technology remains high, is the loss of access to resources that are central to its practice. Technologies dependent on long-distance supply chains and scarce raw materials are susceptible to this form of loss. This can lead to either local or system-wide collapse of technological systems, depending on the degree of interdependence among different elements. On the other hand, supply-driven loss is less likely in technological systems with ample local resources. A less direct, but clearly related model is one in which long-distance exchange of goods generates the wealth necessary to support a particular industry through patronage of attached specialists, or by providing a market for its products. The breakup of these exchange networks would then lead to the abandonment of the industry and the loss of technology, possibly even if the raw materials themselves are locally available.

Demographic factors are a second cause of technological loss, one that has been a particular focus of evolutionary approaches to cultural transmission^17,33–35^. Henrich’s model proposes that when a population of practitioners is very small, the dynamics of imperfect transmission tend to lead to a decline in technological expertise, eventually dwindling to complete abandonment^33^. Complex technologies—specifically, those that are more time-consuming to learn—are potentially more susceptible loss under these conditions. This model has been criticized both with respect to its theoretical foundations, and its applicability to the most widely cited case study, that of pre-contact Aboriginal Tasmania^9,36^. Nevertheless, demography has been suggested as an important factor in other archaeological cases of delayed innovation^32^ and technological loss^17^. As gold mining and metallurgy were specialized crafts involving complex thermodynamic and mechanical transformations, it is worth considering whether demographic changes might have impacted its transmission.

Also falling under the category of demographic drivers of technological loss are factors such as population replacement. Populations are rarely hermetically sealed, and movements of people into, or out of a region may disrupt the vertical transmission of technological traditions in these areas. Just as the movement of new peoples into a region can introduce new technologies, technological traditions may disappear in areas where indigenous practitioners are displaced, killed, or compelled to abandon traditional practices. At the same time, it is worth noting the capacity for adaptation, persistence, and resistance well-documented among Indigenous cultural and technological traditions in the colonial Americas^19,37,38^, suggesting that extreme demographic pressures alone are not sufficient conditions for technological loss.

Social factors driving technological discontinuance include the dissipation of social demand and the active rejection of technological practices. Just as the spread of innovations depends on social and cultural context, social changes can inhibit the inter-generational transmission of technical knowledge and the maintenance of technological traditions. This is particularly true for technologies that are deeply intertwined with particular social or political institutions^39^. Technologies practiced by attached specialists, which are administered or supported by such institutions, may be lost when these instititions collapse or rejected when a preexisting social order is overthrown. Indeed, among archaeologically documented examples of technological loss, there is often a strong correlation with such socio-political turmoil^40^.

It is important to stress that resource restrictions, demographic factors, and shifts in social demand are not independent from one another. Indeed, they can be deeply intertwined. Lack of social demand can decrease the number of practitioners, exacerbate demographic issues with technological transmission, and create supply-chain problems. Likewise, sustained breakdown of exchange networks can change the nature of social demand, as alternative materials and technologies grow in popularity, delaying revival even when supply routes are restored. Demographic changes, such as the replacement of elite strata through invasion, can impose new social pressures on the use or avoidance of certain technologies, especially if those technologies are tightly integrated with elite modes of authority construction.

These different factors impact the archaeological record in ways that are discernible through large-scale analysis of spatial and chronological data, making it possible to test different models of technological discontinuance. In the Caucasus case, if supply factors were the dominant factor in technological loss, one would expect to find both that the availability of gold deposits varied significantly between regions that did and did not experience a decline in goldworking, and that gold use declined most precipitously in areas farthest from the deposits. Demographic changes may be reflected in the quantity of settlement sites, or evidence (genetic and/or archaeological) for the substantial influx of new groups of people. Indirect proxies for loss driven by demographic constriction include parallel decreases in metallurgical scale and sophistication among other industries, or evidence for population contraction, such as a decrease in the number of settlements.

## Results

The assembled database consists of 4555 gold objects from 89 sites, dating between 4000 and 500 BC (Supplementary Datasets S1 and S2; Supplementary Table S1). Objects range from simple sheet beads to complex composite objects incorporating elaborate technologies of filigree, granulation, repoussé, lost-wax casting, and inlays of carnelian, glass, and other materials. The vast majority are excavated objects with some contextual information, primarily from burials.

### Chrono-spatial patterning

**Figure 1:**
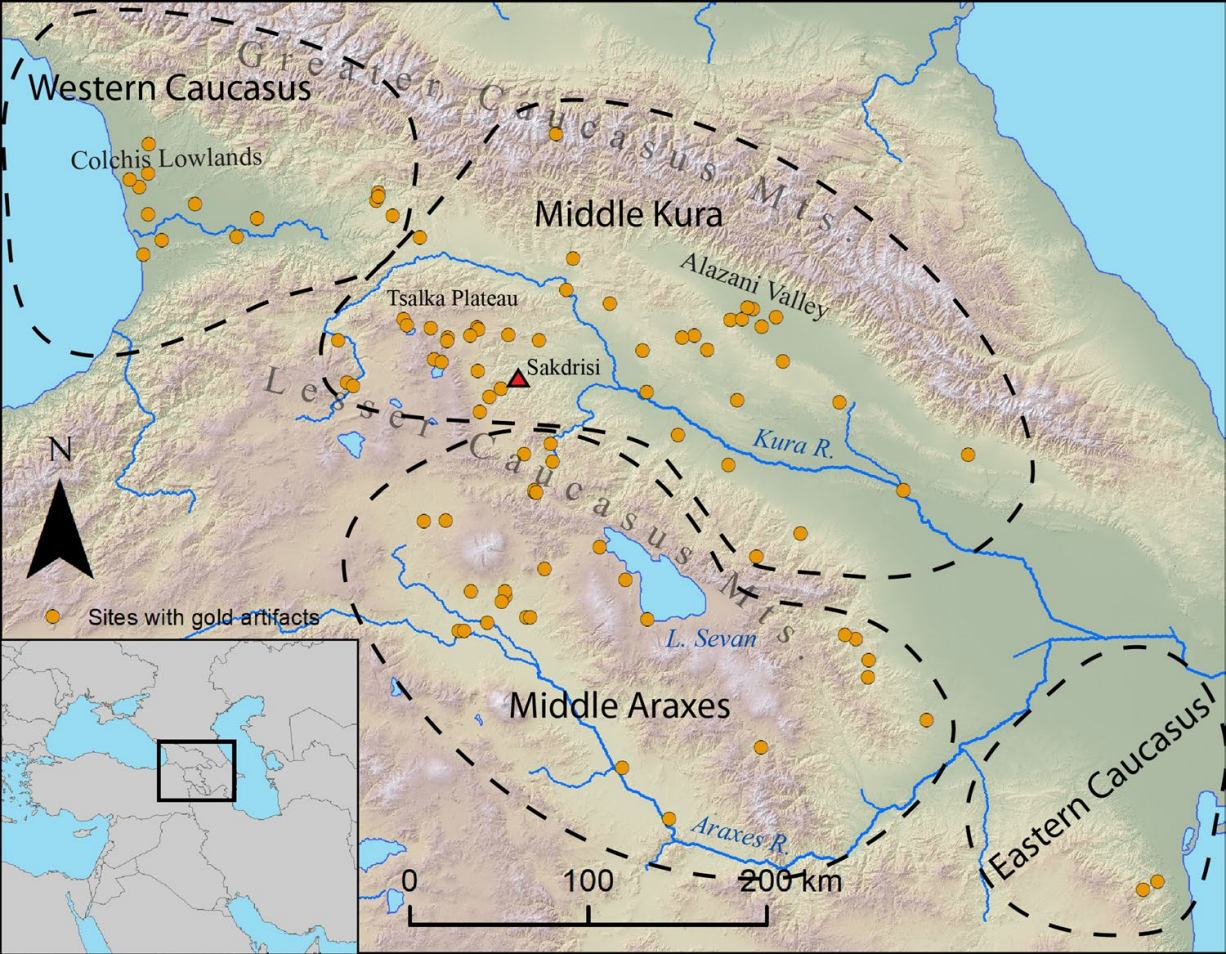
Map of the South Caucasus showing sites with gold artifacts and geographic zones used in the subsequent analysis. The Sakdrisi Early Bronze Age gold mine is indicated by a red triangle.

Objects were assigned to four chronological periods and four spatial zones corresponding to the region’s topography and cultural geography (Fig. 1). The Middle Kura zone encompasses Kura and Alazani river valleys as well as the northern Lesser Caucasus highlands, while the Middle Araxes zone consists of the Ararat Valley and the southern Lesser Caucasus highlands. The Western Caucasus encompasses areas adjacent to the Black Sea, while the Eastern Caucasus zone lies adjacent to the Caspian Sea.

**Figure 2:**
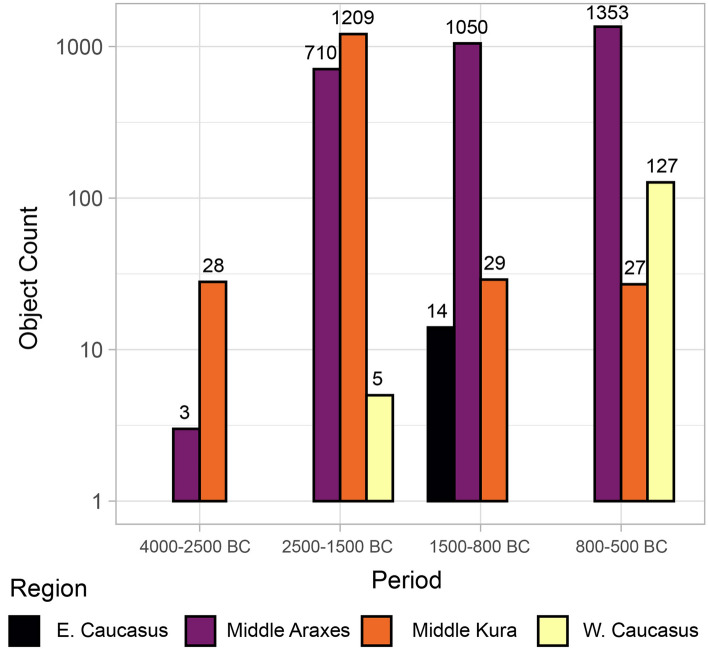
Log-scale plot of gold quantities for different areas over time.

**Figure 3:**
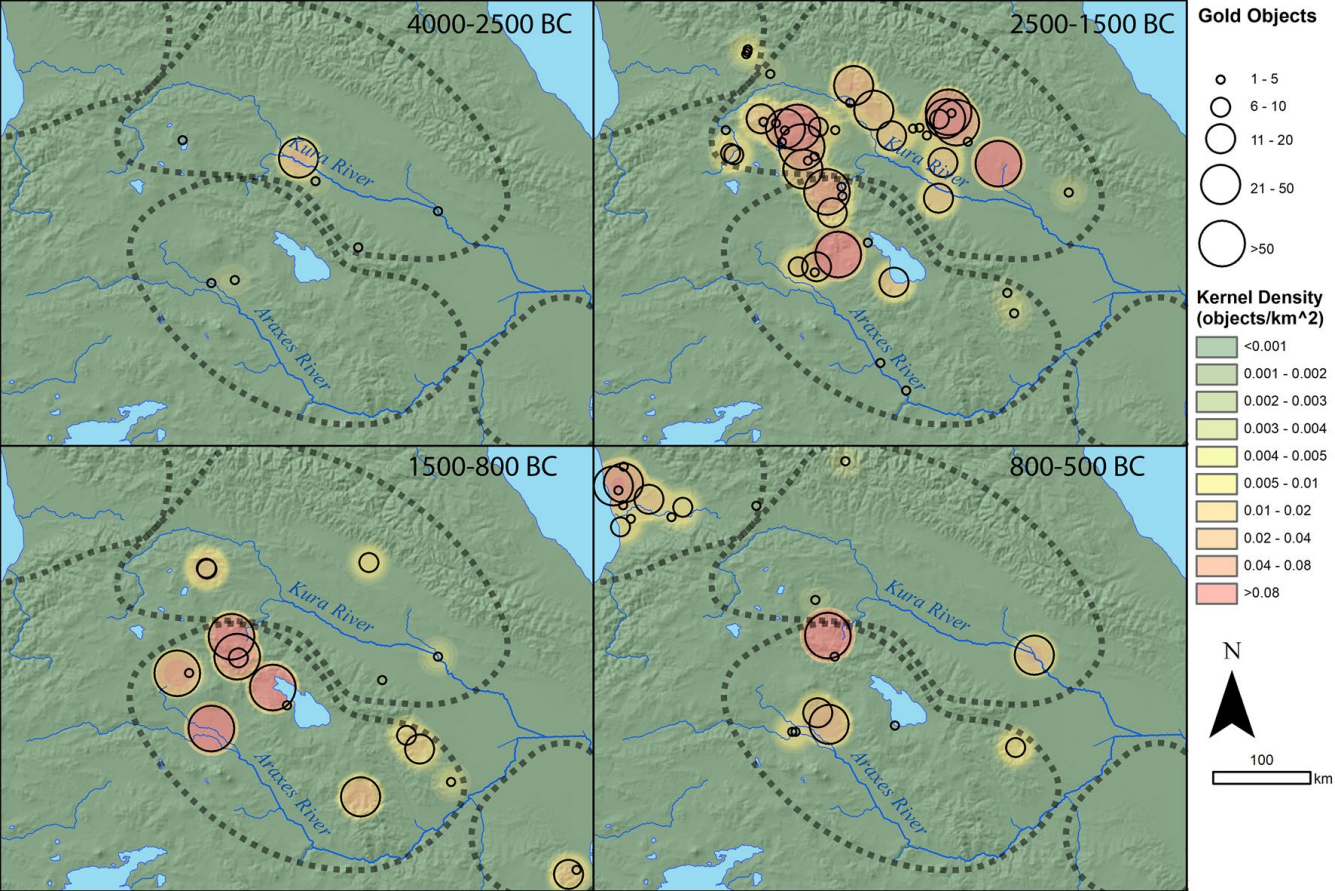
Spatial map of gold use over time. Black circles marking site locations, scaled to the number of gold objects, are overlain over a kernel density raster illustrating areas of high densities of gold objects. Dashed lines correspond to geographic zones in Figs. 1 and 2.

Gold first appears in relatively small quantities in the early 4th millennium BC (Late Chalcolithic) in the Middle Kura zone^41^ (Figs. 2 and 3). Gold objects are relatively scarce in the Early Bronze Age (mid-4th to mid-3rd millennium BC), but the site of Sakdrisi (Middle Kura zone) attests to local mining and production before 3000 BC^27^. Though outside the geographic scope of the database, significant quantities of gold are known from the 4th millennium BC in the North Caucasus as well^28^. Together, these represent one of the earliest gold metallurgical traditions in the world.

From the 2500–1500 BC (Middle Bronze Age), both the Middle Kura and Middle Araxes zones witnessed a massive expansion in gold usage (1919 objects from 48 sites), representing the pinnacle of Bronze Age gold metallurgy across the South Caucasus. Small quantities are also found in the Western Caucasus zone. Complex techniques such as granulation, filigree, and inlay appear, along with the use of gold sheathing for larger objects of organic materials. Exceptional objects—not seen in the rest of the Bronze Age—include a silver bucket with gold trim, gold and silver cups with figural relief decoration and inlay, as well as a cast lion figurine.

For the period 1500–800 BC (Late Bronze and Early Iron Age), the data reveal a stark bifurcation in the technological trajectories of Middle Kura and Middle Araxes zones. Gold use in the Middle Araxes zone remains relatively stable in terms of object quantities, numbers of sites with gold objects, and gold-working technologies. By contrast, the Middle Kura zone shows a decrease of two orders of magnitude, from 1209 objects to 29, with the number of sites with gold artifacts dropping one order of magnitude, from 34 to 5. Many areas with elaborate inventories of Middle Bronze Age gold artifacts have virtually none in the Late Bronze and Early Iron Age.

Crucially, the drop in numbers of gold artifacts is not due to a lack of Late Bronze Age sites or a greater research focus on sites of earlier periods. Thousands of excavated graves date to 1500–800 BC, and settlements are far more common than in the preceding period. Changing patterns of mortuary deposition are unlikely to have caused this apparent decrease. While it is true that the size of graves decreases during this period, material assemblages in 1500–800 BC graves could hardly be considered scant. Late Bronze Age societies clearly had no reservations about depositing elaborate metalwork and other crafts in graves, and non-mortuary metal depositional assemblages also lack goldwork (see further discussion in Supplementary Information). Beads of carnelian, bone, clay, paste, and antimony are all known, but gold, once common, is now extremely rare^42,43^.

The technological complexity and formal diversity in gold objects also declines in the Middle Kura zone, again hinting that the pattern is not simply the result of people choosing not to deposit gold in archaeologically visible ways. Of the few Late Bronze and Early Iron Age gold objects in the Middle Kura zone, most are simple sheet beads, occasionally with incised decoration. Only one site in this area has evidence of granulation technology^44^.

Patterns shift again by 800–500 BC (Iron Age). The Middle Araxes zone continued to yield significant quantity of goldwork, with influences from the Kingdom of Urartu, which expanded into the Ararat Valley at this time. Gold quantities remain low in the Middle Kura zone, though new artifact types appear. A new gold-working center emerges in the Western Caucasus, presaging the brilliant gold-working tradition of Classical and Hellenistic Colchis, mythologized by the Greeks. Though not covered in the database, by the mid-late 1st millennium BC, elaborate goldworking traditions unequivocally reemerge in the Middle Kura zone^45^.

### Modeling access to gold Deposits

**Figure 4:**
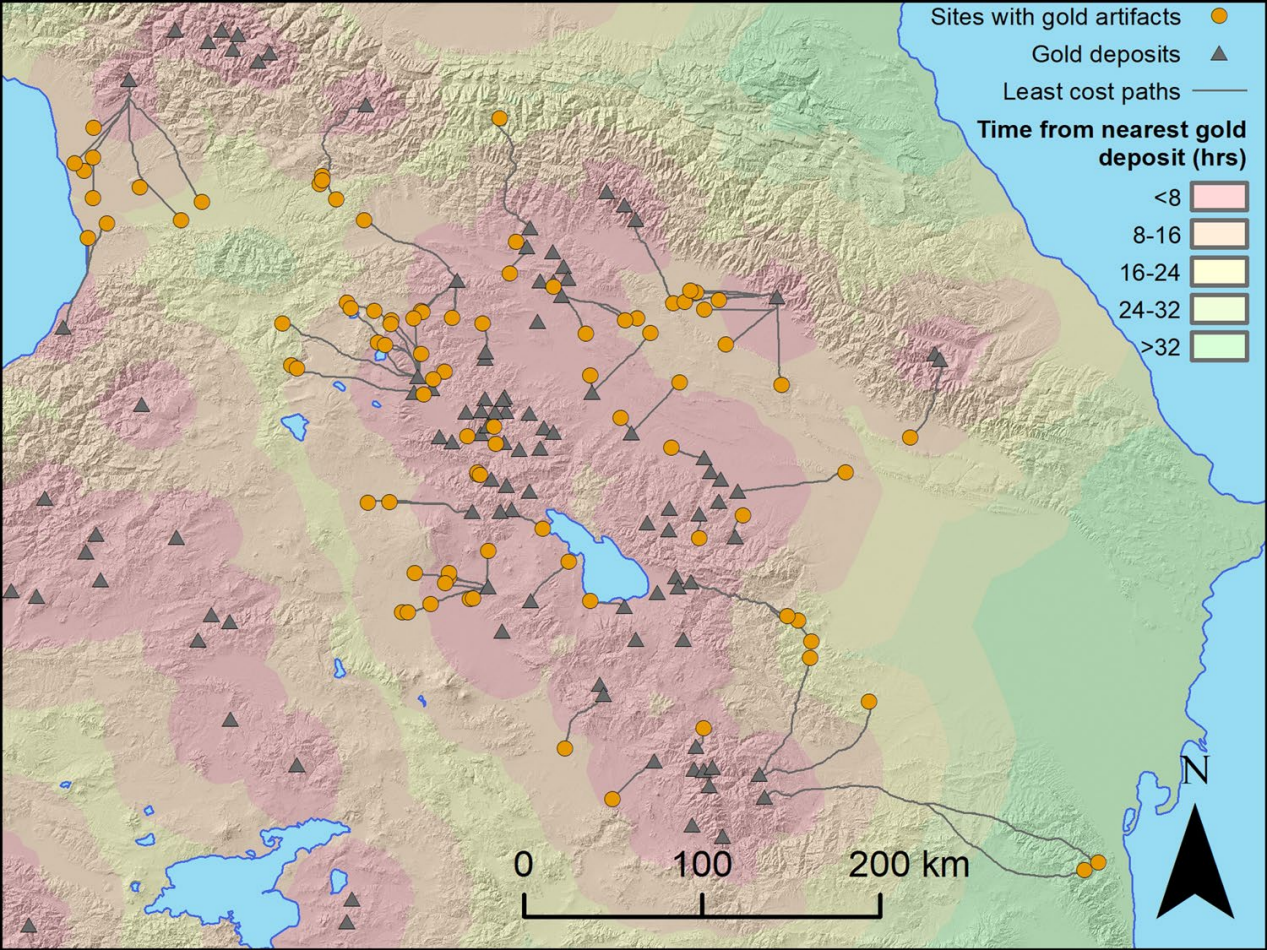
Map of gold deposits, gold findspots, and least cost paths from the nearest deposit to each findspot.

**Figure 5:**
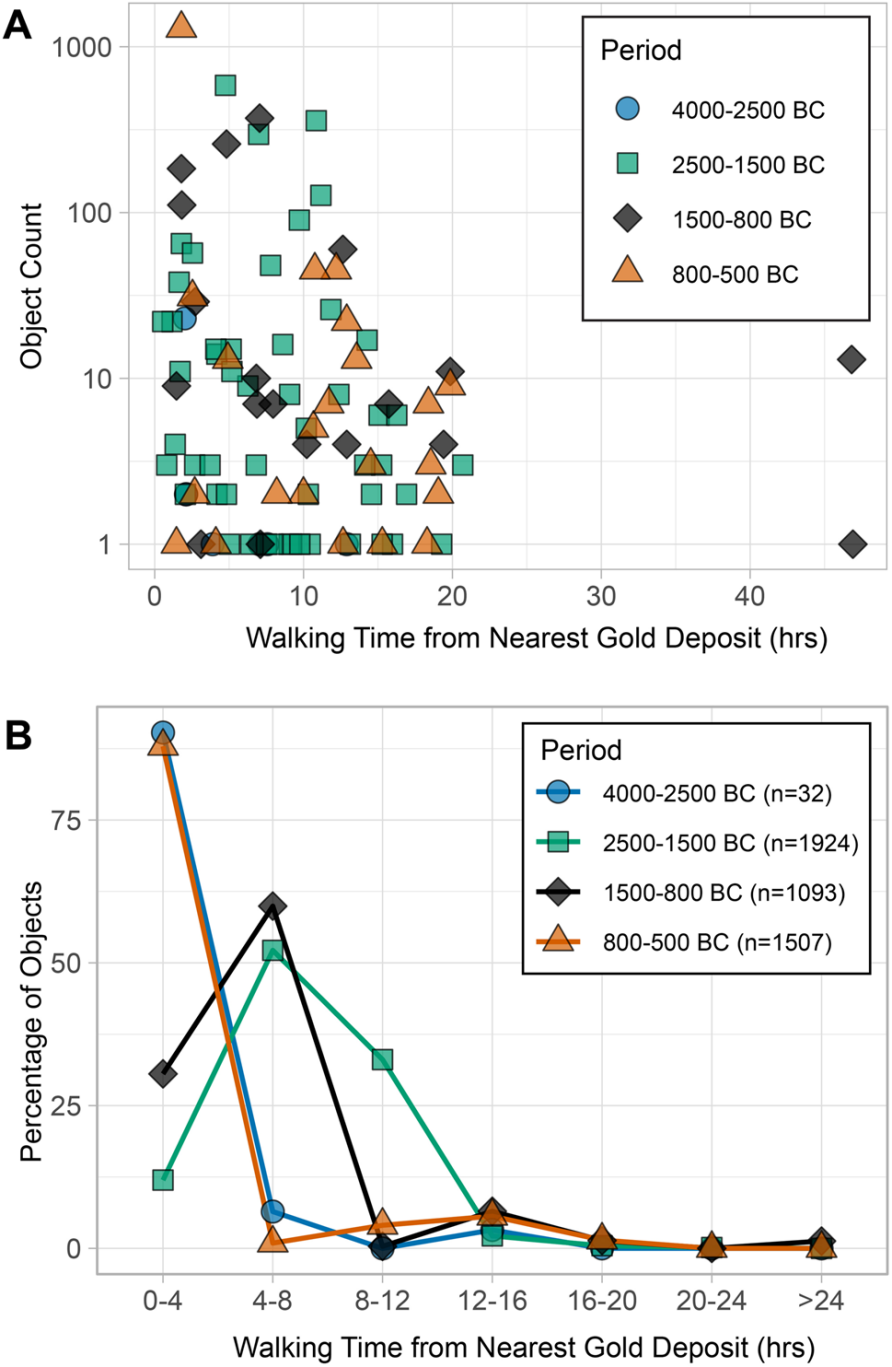
(**A**) Log-scale plot of object numbers vs. least cost walking time to nearest ore deposits, for each period at each site. (**B**) Log-scale plot of percent of objects (of the total number for each period) vs. distance from ore deposit, binned in 4-h increments of least cost walking time from the nearest deposit.

Modeling access to gold resources using cost path analysis and spatial data on gold deposits reveals several key patterns. Gold deposits are widely distributed across the region (Fig. 4). Eighty-seven percent of sites with gold finds accounting for 99% of objects are within two days (16 h) walk from a gold source (Fig. 5A). While direct archaeological evidence for Bronze Age gold mining is sparse and some deposits may not have been mined, it is likely that many more deposits were exploited than the tiny fraction at which Bronze Age gold mining is securely documented. Importantly, access to gold resources did not differ significantly between the Middle Kura and Middle Araxes zones. While the average least cost travel time from the nearest gold source was slightly higher for the former (8.2 h, n = 44) than the latter (7.6 h, n = 29), the difference in the distributions is not statistically significant (Mann–Whitney U-Test, U = 532, n_1_ = 29, n_2_ = 44, p = 0.236). Anecdotally, it is perhaps worth noting that among Middle Bronze Age sites with more than 100 gold objects, the three from the Middle Kura zone (Zilicha, 10.9 h; Trialeti, 7.0 h; Ananauri, 11.2 h) are farther from an ore deposit than the one from the Middle Araxes zone (Karashamb, 4.7 h). Given the methodological considerations against prioritizing individual gold-rich sites, which at least partly a product of archaeological happenstance, over the broader pattern, however, one should not overinterpret this feature of the data.

Plotting object quantities against least cost walking time from the nearest ore deposits for different periods (Fig. 5), provides a way of visualizing how quantities of gold vary in relation to proximity to a gold deposit. Two features are immediately apparent. First, as might be expected, all periods show a rough trend towards decreasing quantities of gold as walking times from deposits increase. Second, the fall off curves (Fig. 5B) drop rapidly as a function of walking time because ore deposits are widespread. Except for a divergence at the 8–12 h point, the fall off curves for 2500–1500 BC and 1500–800 BC are largely similar. Examination of this point of divergence with reference to the un-binned data (Fig. 5A) shows that it is largely driven by least cost time differences of about 3 h for two or three sites with large numbers of gold artifacts, mostly beads (see also Supplementary Fig. S6). Given the approximations involved in the gold access modeling and the methodological cautions against privileging sites with exceptional quantities of gold, one should not overinterpret this feature. The general similarity of these curves suggests that the pattern of access did not radically change between these two periods.

In a model of technological loss where a breakdown in long distance trade limited the availability of gold in areas far from deposits, one might expect areas closer to ore deposits to experience a proportionally smaller decline in gold quantities than those farther away. Calculations of the absolute and relative (%) declines in gold between the Middle Bronze Age (2500–1500 BC) and Late Bronze/Early Iron Age (1500–800 BC) among sites at different least cost time intervals from gold deposits shows that this is not the case (Table 1; Supplementary Dataset S1). Relative (%) changes are particularly instructive, as this corrects for the fact that absolute object counts are much lower for time bins farther from gold deposits. In the Middle Kura Zone, steep drops of 89–100% are recorded across all time intervals, illustrating how the decrease is present in both areas closer to deposits and those farther away. The relative decline is constant (and severe) across all least cost time intervals: there is no evidence that declines within the Middle Kura zone are mitigated according to proximity to gold sources. Though the values for the absolute and relative changes are different for the Middle Araxes Zone, in both regions, there is no correlation with least cost time from deposit. While in the Middle Kura zone, the relative decline is effectively unchanging as a function of time from deposit, in the Middle Araxes Zone, there is a mixed pattern of increases (0–4, 4–8, 12–16, and 16–20) and decreases (8–12 and 20–24 h intervals) of variable magnitude that also lacks a clear relationship to time-from-deposit. Thus, in both these regions and indeed for the South Caucasus as a whole, the walking time from an ore deposit is a poor predictor of what areas witnessed the steepest declines in gold quantities. By contrast, these data underline how patterns of decline *are* dependent on region: overall, the Middle Kura zones witnessed an 98% decrease, while the Middle Araxes zones experienced a 48% increase.

**Table 1:** Change in gold quantities between the periods 2500–1500 BC and 1500–800 BC among sites at different least cost distances from gold deposits for the Middle Kura zone, Middle Araxes zones, as well as the entire database, expressed as both absolute and relative (%) change. Note that for both zones, distance from deposit has no bearing on the relative change, but the patterns differ strongly between regions. ^a^Percent change is undefined (effectively infinite) because number of objects from 2500–1500 BC is 0. ^b^Percent change undefined because number of objects from both 2500–1500 BC and 1500–800 BC is 0.

Least cost time from deposit (walking hours)	0–4	4–8	8–12	12–16	16–20	20–24	> 24	Overall
Middle Kura (objects)	− 148	− 365	− 627	− 33	− 7	0	0	− 1180
Middle Kura (% change)	− 99%	− 94%	− 100%	− 89%	− 100%	–^b^	–^b^	− 98%
Middle Araxes (absolute)	+ 252	+ 16	− 5	+ 67	+ 13	− 3	0	+ 340
Middle Araxes (objects)	+ 311%	+ 3%	− 56%	–^a^	+ 650%	− 100%	–^b^	+ 48%
All regions (objects)	+ 104	− 349	− 632	+ 29	+ 6	− 3	+ 14	− 831
All regions (% change)	+ 45%	− 35%	− 99%	69%	67%	− 100%	–^a^	− 43%

## Discussion

The large-scale geospatial analysis of Caucasus gold objects reveals a comprehensive decline in gold use—by approximately two orders of magnitude—during the Late Bronze Age in the Middle Kura zone. While this pattern has been occasionally noted in excavation reports covering localized areas ^46–48^, the scale of the analysis shows unequivocally that this is a genuine phenomenon, not an artifact of patchy archaeological research. It is important to note that the archaeological record of gold objects mostly consists of intentionally deposited artifacts in graves (Supplementary Table S1), and is therefore subject to selective processes that can distort how social use patterns appear the archaeological record. However, the Late Bronze Age decline in gold does not correlate with an overall decline in the deposition of metalwork, or an avoidance of items of personal adornment in mortuary assemblages. The absence of gold also extends to non-mortuary depositions of metalwork in the Middle Kura Late Bronze Age ^49^. Moreover, the continued visibility of gold in the mortuary record of the Middle Araxes zone, which shares many aspects of material culture with Middle Kura zone, suggests that if gold were circulating in the latter region, it would appear in burial assemblages. These observations suggest that it is unlikely that significant amounts of archaeologically invisible gold were circulating in the Middle Kura zone. Despite the early adoption of gold metallurgy, pioneering complex and laborious hard-rock gold-mining technologies, and an elaborate, prolific gold-working tradition, societies in the Middle Kura zone largely abandoned gold metallurgy for 700–1000 years, in marked contrast to areas further south. Integration of archaeological and geological data within this geospatial framework allows us to test various causal models for this phenomenon.

The resource-restriction model proposes that the elimination of access to key resources, either through a breakdown of exchange networks or the exhaustion of mines at about 1500 BC precipitated the loss of gold metallurgy. The widespread distribution of gold deposits suggests that the model of exchange network breakdown is unlikely. Areas like the Middle Araxes Zone that maintained a stable gold metallurgical system through the Late Bronze Age are not significantly closer to known gold deposits. Moreover, within the Middle Kura zone, areas that are closer and farther from gold deposits both experienced the same decrease in gold usage. Finally, there is no broader archaeological evidence for decreased contact within or between the regions in question, as both the Middle Kura and Middle Araxes zones belong to the same broad material culture horizon in the period 1500-800 BC.

The question of local finite resource exhaustion is more difficult to address. As is the case in most regions of the world, direct evidence for Bronze Age gold mining is limited. The chronology of mining activity at the best-documented Caucasus gold mining site, Sakdrisi, does not correspond well with peaks in gold usage. Samples from mining shafts yielded radiocarbon dates between c. 3300 and 2600 BC, but none that correspond to the Middle Bronze Age region-wide gold-working apogee at c. 2500–1500 BC ^27^. This suggests that a significant amount of Bronze Age gold mining has yet to be detected archaeologically, perhaps because it involved difficult-to-identify placer mining.

Exhaustion of certain gold mines could theoretically have led to local declines in gold usage, especially in areas with somewhat fewer nearby alternative options (for instance, the sites in the lower Alazani Valley). However, existing evidence about each deposit’s size, and ease of extraction, and more importantly dates of exploitation is too fragmentary to provide robust support for this hypothesis at present. In areas such as the Trialeti Plateau, close to the very large and productive deposits in southern Georgia ^50^, the theory of local resource exhaustion would raise the question of why such technically proficient gold miners and prospectors were unable to locate one of the many other gold deposits available nearby. Abundant gold sources in the region are plausible evidence against source-exhaustion as the driving factor in technological loss. Further research on early gold mining may allow us to replace some of the model’s assumptions and approximations with concrete information, but the intensive research required to identify and date Bronze Age gold mining activity means that a reasonably comprehensive regional-scale spatiotemporal picture of Bronze Age gold mining is a long way off.

The demography-driven loss model might predict that declining populations, or displacement by invaders might lead to a breakdown in intergenerational transmission of technologies. Two methods of assessing whether the population was under demographic constriction of the kind that might cause technological loss are comparisons with related crafting technologies, and general evidence for population change. Abundant evidence shows that specialized metallurgical traditions—other than gold—were actively maintained and expanded during the period 1500–800 BC in the Middle Kura zone. A notable feature of the abandonment of gold metallurgy is that it diverges so strongly from other metallurgical traditions in the very same area. In terms of both scale and complexity, bronze metallurgy thrived during this period, with iron metallurgy becoming more widespread after 1000 BC ^49,51,52^. Other specialized decorative crafts, including bead manufacturing, using carnelian, bone, and vitreous paste, also flourished ^42^, suggesting that skilled crafting traditions were not demographically constrained in ways that might lead to technological loss.

From a more general demographic perspective, the proliferation of settlements, fortresses, and graves during the Late Bronze Age reveal, if anything, a trend toward population *growth* as previously mobile populations aggregated around fortified strongpoints ^53–57^. Late Bronze Age settlement sites are far more numerous than those of the Middle Bronze Age across both the Middle Kura and Middle Araxes zones. Likewise, there is little evidence for major demographic displacement during the Middle-Late Bronze Age transition; available genetic evidence does not show major turnover in the South Caucasus at this time ^58,59^, and archaeological data provide little evidence for exogenous drivers of the Middle to Late Bronze Age social transformations. In combination, the intensification of other specialized craft traditions and the evidence for population expansion suggest that demography-driven limitations on technological transmission were not a primary factors in discontinuance of gold metallurgy. Indeed, the expansion of other complex craft traditions suggests that gold metallurgy was not passively lost, but actively rejected.

Both resource restrictions and demographic factors are unsatisfactory as primary explanations for the decline of the gold metallurgical tradition in the Middle Kura zone. In contrast, a social model of active rejection, emerging from the social and political transformations in the mid-late 2nd millennium BC, provides a more plausible mechanism. In the Middle Kura and Middle Araxes zones, the period 2500–1500 BC is characterized by a marked increase in social hierarchy and a steep decline in permanent settlement, reflecting a transition to a greater dependence on mobile pastoralism ^55^. Large kurgans with richly-furnished burial chambers appear, in some cases reaching over 100 m in diameter and more than 10 m high ^60^. In contrast, the period 1500–800 BC in the Middle Kura zone is characterized by a more muted attitude toward displays of elite material wealth in the burial record. Enormous kurgan burials, hallmarks of the previous period, are absent, and graves, even ones with substantial burial inventories, are smaller. Fortresses, rather than large kurgans, are the most prominent monumental constructions, and the largest and most substantial of these, with massive cyclopean walls, are not as common in the Kura River lowlands as they are farther south and west ^55^. The social organization of fortresses communities is much discussed ^53,61,62^, and it is not yet clear whether fortresses reflect the translation of the Middle Bronze Age social hierarchy from the burial environment to the built environment of living communities, or whether they represent a turn away from the more extreme patterns of social hierarchy. While rich graves do occur in the period 1500–800 BC in the South Caucasus ^63,64^, in many areas there is a deemphasis on mortuary display of extreme hierarchy in the Late Bronze Age relative to the preceding period, which some have suggested reflects a “social leveling” process ^48^. This manifests not only in the decrease in gold in the Middle Kura zone, but also decreases in the size of mortuary monuments (both kurgan mounds and the burial chambers within them) and the richness of their furnishings.

Compared to contemporary Late Bronze Age palaces in the Eastern Mediterranean and Near East, or the even later Iron Age Urartian fortresses in the South Caucasus, Late Bronze and Early Iron Age fortresses, while impressive in their monumental defensive fortifications, contain relatively little evidence for elite residences or large-scale centralized administrative facilities. Fortresses do seem to be important centers for the production of institutional authority ^61^, but the extent to which that authority was vested on individual rulers or more communal governance structures remains unclear.

Gold was a central element of elite mortuary rituals among South Caucasus societies 2500–1500 BC, featuring prominently in large kurgan burials. The use of gold in goblets, and items of personal adornment, as well as gilded wooden implements and/or furniture, suggest that the material was a key means of expressing, displaying, and reinforcing hierarchy at a time when marked social inequality increased significantly. Given the close association between gold and the Middle Bronze Age social order, it is likely that the Late Bronze Age social traditions, emphasizing communities and institutions over extreme elite display centered on individuals, would reject the material trappings of that earlier socio-political order. Interestingly, the proliferation of fortresses and weaponry ^52^, as well as bioarchaeological indicators of interpersonal violence ^65^, suggests a significant level of peer-polity competition during the Late Bronze Age. Yet the communities of the Middle Kura zone largely chose not to play out this competition through the aggrandizement of individuals, at least, not in the mortuary sphere, and not to the level seen in the Middle Bronze Age.

The result was a decline in the social demand for gold and the abandonment of this technological tradition. It is perhaps significant that one of the few Late Bronze Age burial sites with gold artifacts in the Middle Kura zone (Tsitelgori) is most reminiscent of the earlier traditions of conspicuous consumption in a mortuary context. Kurgan #1, containing three gold beads and four gold “settings”, also included 50 bronze objects and the remains of 13 sacrificial animals ^44^. At the same time, it is worth noting that many similarly elaborate burial inventories from the Middle Bronze Age of the same area (e.g. Ananauri and Tsnori) include much more gold.

Against a background of declining social demand for gold, resource supply and demographic factors may have come into play in a secondary cascade of consequences, intensifying and cementing process of discontinuance, in essence the inverse of the cascade model of invention ^66^. Declining demand would have led to a preference for other metals in mining prospection, placing further constrictions on supply. If craftspeople were attached to and dependent on mobile pastoralist elites of the Middle Bronze Age for patronage, the breakdown of these social structures would undoubtedly have inhibited intergenerational transmission of metallurgical techniques, especially technologies such as granulation and filigree, which were mostly limited to precious metalworking.

A key outstanding question remains: why did the Middle Araxes zone, which shares many aspects of material culture with the Middle Kura zone in the Late Bronze and Early Iron Age, not undergo the same process of technological rejection? Addressing this issue requires comprehensive reexamination of subtle differences in social organization between the regions, but there are hints that traditions of ostentatious elite display persisted into the Late Bronze Age in the Middle Araxes region, at least in some areas ^63^. Clear reasons for the bifurcation in gold use between the Middle Kura and Middle Araxes zones may yet prove elusive—stochastic responses to social transformations, coupled with cascading consequences and path dependence may lead to significantly different outcomes despite similar initial conditions. Regardless of the reasons, the divergence in gold use between these regions shows that even prestigious technological traditions are not automatically maintained.

## Conclusion

Geospatial analysis of a large database of Bronze and Early Iron Age gold overcomes significant methodological challenges facing the study of technological discontinuance, and provides a template for testing models across cultural contexts. Crucially, it shows that social factors play a major role in the collapse of technological systems, and that such systems are not inherently self-sustaining. Furthermore, the analysis indicates that technological discontinuance occurs in a range of circumstances that have not been highlighted by prior empirical or theoretical research. Namely, societies may reject technologies even after widespread adoption if the foundations of social demand and cultural acceptability shift. Importantly, technological discontinuities may not correlate with times of broader social collapse, as shown by demographic expansion and innovation in other crafts coinciding with the precipitous decline in gold-working. Technological discontinuance is far from rare—many innovations result in the discontinuation of earlier technologies. For example, in many regions, the innovation of iron led to the eventual abandonment of bronze as a material making weapons and agricultural tools. The latter process is not a trivial, perfectly synchronized mirror image of the former—they frequently did not happen at the same time, leaving periods of coexistence where bronze and iron forms strongly parallel one another ^67,68^.

The Caucasus case reveals that empirical research on cases of technological loss is essential for building models of technological change and innovation. Though archaeological analyses face significant methodological hurdles, big data approaches provide a genuine path forward, distinguishing broad scale patterns and mitigating issues of happenstance in archaeological discoveries. Ultimately, robust analyses of technological discontinuance build a richer picture of social dynamics involved with the processes of innovation.

## Methods

Data on gold artifacts, including findspots, quantities, dates, and working techniques, were compiled from published sources spanning roughly 130 years of archaeological excavation. The database covers artifacts from the three South Caucasus countries (Georgia, Armenia, and Azerbaijan) and stretches from the earliest gold to c. 500 BC, as very large quantities of Classical-period gold make precise quantification difficult. Modern national and linguistic boundaries, which affected archaeological research even during the Soviet period, posed significant challenges for the assembly of the database, which extend beyond the basic challenge of integrating research published in low-circulation publications in many different languages. Chronological schemes and archaeological cultural attributions vary between Georgia, Armenia, and Azerbaijan, requiring careful consideration. Dating of finds was largely recorded as reported in the source material, though in a small number of cases, mostly relating to excavations before 1950, interpretive adjustments to the reported dates were justified. Objects were assigned to chronological categories broad enough to mask minor differences in regional chronologies, while still capturing the major temporal patterns under study. For the complete referenced database of sites and objects, as well as a thorough discussion of data quality, methodological decisions, and chronological considerations, see the supplementary information.

Methods of data visualization and analysis were calibrated to capture the most significant patterns in the data without overemphasizing discoveries of sites with exceptionally large quantities of gold, which may partly result from archaeological happenstance. This approach involved selecting visualization protocols that emphasized both the numbers of sites, and the numbers of objects found at them. Interpretations based on quantitative variations of one or more orders of magnitude, using data from many sites, were considered most robust. For these reasons, logarithmic scales were favored for object counts. A similar motivation prompted the use of a dual visualization for spatial mapping, overlaying point-size distributions scaled to the number of objects at each site on a kernel density estimation raster, which provides a continuous picture of hotspots in gold usage (Fig. 3). The resulting plots effectively illustrate both numbers of sites with gold and quantities found, making it easier to identify the most significant chrono-spatial patterns.

Modeling of gold supply access was conducted by georeferencing published maps of gold deposits ^27,69^ and calculating least cost paths from the nearest deposit to each archaeological site with gold artifacts, using Tobler’s hiking function on a 3 arc-second digital elevation model (SRTM3; ~ 90 m resolution). Coverage of the gold deposit maps is good for eastern Turkey, as well as much of the three South Caucasus countries, but it lacks information about gold deposits in northwestern Iran and the easternmost parts of Azerbaijan. In practice, this affects least-cost calculations only for the two sites in the Eastern Caucasus zone; they may be closer to gold sources than indicated by the analysis.

Modeling access to gold deposits in this way involves several approximations. Geological gold deposit maps are an imperfect reflection of deposits that were mined in the Bronze Age, and gold found at a site did not necessarily come from the nearest gold deposit. From a social perspective, conflicts and alliances can make distant deposits controlled by an ally more accessible than proximal sources in the hostile territory. Nevertheless, spatial access modeling assesses *potential* access to gold resources, and does not make any claim that such resources were mined at a particular time, or that any particular gold object came from a specific deposit. Two other considerations further justify the approximations in the model. First, placer mining of gold leaves very limited archaeological remains, and modern mining frequently obliterates prehistoric shafts, adits, and galleries. Thus, ancient gold mining was likely much more widespread than the number of archaeologically recorded prehistoric gold mining sites might suggest. Second, archaeological research on early gold mining in the Caucasus has revealed that Bronze Age miners were capable of extracting gold even from underground low-grade hard-rock deposits as early as the late 4th millennium BC ^27^. Except in cases where gold-bearing deposits are very deeply buried, many of these deposits, or secondary placer deposits nearby, would have been potentially exploitable by Bronze Age gold miners. Therefore, on a regional, systemic scale, modern geological maps of gold deposits are an acceptable approximation of what gold deposits were available to metalworkers in different parts of the South Caucasus in the Bronze Age.

## Supplementary Information

Supplementary Information.

Supplementary Dataset S1.

Supplementary Dataset S2.

Supplementary Dataset S3.

## Data Availability

The data and code used in the paper is provided in three supplementary datasets. Dataset S1 is .xlsx file readable by Microsoft Excel. Dataset S2 is contains a geodatabase of vector data and georeferenced rasters readable with Esri ArcGIS software (e.g. ArcGIS Desktop 10.8 https://desktop.arcgis.com/en/). Dataset S3 contains R code for plotting Figs. 2, 5, and S6, the R code for the statistical tests, and .csv files derived from Dataset S1 on which the R code acts. The R files are accessible with RStudio (version 1.1.447 https://www.rstudio.com/), an open-source software.
