# Supplementary material for: Technological rejection in regions of early gold innovation revealed by geospatial analysis
Source: Sci Rep. 2021 Oct 13;11:20255. doi: 10.1038/s41598-021-98514-7 (PMC8514478; doi:10.1038/s41598-021-98514-7)
Supplement: Supplementary file 1 — Supplementary Information. [file 41598_2021_98514_MOESM1_ESM.pdf]

Technological Rejection in Regions of Early Gold Innovation Revealed by Geospatial Analysis

Nathaniel Erb-Satullo[1]

[1] Cranfield Forensic Institute, Cranfield University
n.erb-satullo@cranfield.ac.uk
ORCID: https://orcid.org/0000-0001-6463-3120

Supplementary Information

Database Assembly, Data Quality, and Methodological Details

Assembling and mapping patterns in the development of gold metallurgy requires careful consideration of key methodological challenges. Foremost are questions of archaeological visibility and data completeness. Is gold use in the South Caucasus likely to be reflected in the archaeological record for the relevant time periods? Are finds of gold likely to be noted and published in excavation reports, and what fraction of gold artifacts have been published in enough detail for inclusion in the database?

In defining the scope of the database, both chronological and geographical factors were carefully considered. Given the focus on the phenomenon of technological abandonment in the Middle Kura area, a decision was made to restrict the geographic scope of the database to the three modern-day countries of the South Caucasus, while acknowledging that there are some spectacular gold finds that lie just outside this zone, in the Northern Caucasus (Sagona 2018:163-168), and in northwestern Iran (Danti 2014; Wilkinson 1965). The density of archaeological research in the South Caucasus, particularly in modern day Georgia and Armenia, makes assessments and interpretations, most crucially those related to the *absence* of gold, more robust. In comparison, neighboring areas of northeastern Turkey have had far less archaeological research,

though limited work has demonstrated close connections with the South Caucasus (Sagona 2000; Yardimiciel et al. 2018). Chronologically, the database spans from the late 4th millennium BC, when the earliest gold appeared in the region, to c. 500 BC, the very beginning of the Classical period. This latter cut-off was chosen because gold is found in such huge quantities after 500 BC that quantification is difficult: exact numbers of objects are typically not reported. For example, a single grave at the Classical-Hellenistic sanctuary at Vani (one of many gold-rich graves at the site) contained more than 1000 gold objects (Kacharava 2005:293; Kacharava and Kvirkvelia 2008).

The appropriate chronological resolution for exploring the ebb and flow of gold metallurgy was another important methodological decision (**Supplementary Fig. S1**). Sources vary with respect to the precision with which the remains were dated, while regional chronologies and cultural designations differ with respect to the details. Radiocarbon dates from sites and contexts containing gold are so rare as to negate the possibility of doing more complex chronological modelling seen in other studies (Palmisano et al. 2021). Fine-grained chronological designations relying on more subtle differences in material culture may hold only for the local regions in which they developed. It was beyond the scope of this project to reevaluate the chronology of each of the sites in the database, so the chronological information given in the publications was largely reported as described in the publication, except in a small number of instances, frequently in excavations before 1950, when adjustments or interpretations were necessary. Original source chronologies were noted in the database, but a relatively coarse-grained chronological categorization was developed for mapping and other data analysis purposes. For this reason, mid-late 3rd millennium remains belonging to the so-called "Early Kurgan Cultures," which are variously assigned to the end of the Early Bronze Age or the beginning of the Middle Bronze Age (Manning et al. 2018; Sagona 2018:305ff), and the more securely Middle Bronze Age cultures of the first half of the 2nd millennium BC were grouped together. Gold use practices of the Early Kurgan cultures are more closely aligned with those of the Trialeti Culture (early 2nd millennium BC) than those of the Early Bronze Age Kura-Araxes Culture, so the designation as Middle Bronze Age seems most appropriate here, as least as a heuristic. In combination, the period 2500-1500 BC represented the pinnacle of Bronze Age gold metallurgy across

the region. More fine-grained relative assessments of gold abundance within the period 2500-1500 BC would be difficult to make, as chronological relationships are much debated, and there are relatively few radiocarbon dates (Kavtaradze 1983; Puturidze 2003; Rubinson 2013). Similarly, Late Bronze through Iron I (c. 1500-800 BC) sites in the database were grouped together, though at times the sources are chronologically more specific. However, because the period 800-500 witnessed a new beginning for gold metallurgy in some areas, presaging the spectacular resurgence of Classical and Hellenistic goldworking in the Western Caucasus (Colchis) and Middle Kura zones, these materials were classified separately, to capture the beginnings of this transformation.

These broad categorizations largely avoid issues of chronological differences in the literature, while capturing the most significant patterns in the adoption and rejection of gold metallurgy. More fine-grained chronological divisions, because they reduce the number of sites per period, may produce patterns that are more reflective of the luck of archaeological discovery rather than a genuine reflection of ancient gold use.

The overwhelming majority of gold artifacts in published literature come from mortuary contexts. The burial record for the periods between 2400-1500 BC and 1500-800 BC, the two key periods under consideration, is very robust, with excavated graves dating to these periods estimated to be well into the thousands. The interpretive problems of relating mortuary assemblages to non-mortuary lifeways are well known (Flad 2002). Objects deposited intentionally in hoards or graves undergo a selective process, so it is not always easy to link patterns in such intentional deposits with value systems and usage patterns among the living (Geselowitz 1993:240; Needham 1988).

In one sense, the question of whether changing depositional practices may skew the archaeological picture is difficult to resolve, and one faced by study of any class of material culture that tends to be found mostly in intentionally curated deposits. In the South Caucasus, the overwhelming majority of gold finds are found in mortuary contexts. making it difficult to compare, for example, the decline in gold use across different depositional contexts. However, there are several features of mortuary and non-mortuary depositional patterns in the South Caucasus Bronze Age that suggest that the lack of gold in the LBA-EIA Middle Kura zone is *not* due to an archaeological visibility issue. First, it is worth noting that depositing metal artifacts and other ornaments in graves is a common

practice throughout the periods under discussion, including in areas where gold use declines in the Late Bronze Age. Thus, the decline in gold seen in the Middle Kura zone is *not* part of an overall decrease in the visibility of material goods, including metalwork, in the mortuary record. Secondly, gold is also lacking among non-mortuary metal deposition contexts in the Late Bronze/Early Iron Age in the Middle Kura zone. No gold artifacts are reported from the sanctuary sites at Nazarlebi and Shilda (present-day eastern Georgia), despite the fact the sites yielded hundreds of ritually-deposited bronze objects (Bukhrashvili et al. 2019; Maisuradze and Inanishvili 2006). Widespread deposition of metalwork in hoards is a more common practice in the Western Caucasus zone during the period 1500-800 BC, but it is worth noting the absence of gold in hoards of this region as well (Akhvlediani 2005; Lordkipanidze 2001). The fact that the general absence of gold extends to both mortuary and non-mortuary kinds of metal deposition in the Middle Kura zone shows that the absence of gold does not simply extend to one kind of depositional behavior. One could theoretically envision a scenario in which a Late Bronze/Early Iron Age cultural tradition emerged in the Middle Kura Zone, in which gold was still widely used and valued, but, despite a clear willingness to deposit other valued materials, including metals, in a range of different, archaeologically-visible contexts, gold was somehow deemed inappropriate for such uses. Such a scenario, however, stretches the bounds of plausibility.

As in many areas of the world, the pace of archaeological publication means that many excavated assemblages remain incompletely published. In terms of reporting, however, the overall rarity of gold and its present-day prestige means that, when they are found, gold finds are more likely to be described and illustrated in excavation reports, discussed in other publications, and displayed prominently in museum collections. In this respect, therefore, gold has an advantage as a material for quantitative synthetic analysis: its “visibility” in the archaeological literature, even when excavations are incompletely published.

A related concern in quantitative mapping is the unevenness of archaeological investigation in different regions of the South Caucasus. While every effort has been made to record all known gold finds falling within the spatial and chronological limits of the database, the assembled database includes some apparent gaps. Some areas, such as

eastern and northeastern Azerbaijan, have less robust coverage, likely due to a combination of limited archaeological research and perhaps also to a lack of gold use in these areas. However, the density of published research in areas relevant to the question of technological loss is more than sufficient for the aims of this paper. Thus, the assembled database is likely an accurate reflection of archaeologically recovered gold, and ultimately, ancient use patterns.

During the process of assembling the data, it rapidly became clear that some objects have been discussed in multiple publications. For unique and exceptional artifacts, this is easily recognized, but there are other cases of ambiguity. The database was checked for duplicate entries and clear cases of duplication eliminated. Counts and descriptions from primary excavation reports were favored over secondary summaries wherever possible. For a very small number of objects in the database, duplication was possible but could not be demonstrated by a preponderance of evidence. Overall, however, issues of possible duplication are minimal and do not affect the broader patterns.

Some reports do not quantify numbers of objects. Where photographs are given, it was possible to count objects, but where objects like beads are piled upon one another for the photograph, such numbers may be undercounts. Where no images are published, but the objects are clearly referred to in the plural, objects are entered in as two objects, and this decision was noted in the "additional notes" column of the database. Similarly, it was sometimes difficult to tell whether two photos are multiple views of a single object, or two very similar objects. When in doubt, the policy has been to err on the side of undercounting.

A final quantification consideration is how to count artifacts containing many separate pieces. For example, should a necklace containing 30 beads be counted as a single object or as 30? Counting beads individually has the effect of greatly increasing artifact counts from sites with necklaces (e.g. Loriberd), which might seem to overemphasize their significance relative to a burial with a single, yet exceptional object like a gold cup. While not a perfect solution, several considerations prompted me to treat beads and fragments of gold sheet as separate items. First and foremost, it represents the most consistent, verifiable, and repeatable approach to classifying objects. There is often

uncertainty about whether a collection of beads that are published together on a string were in fact originally part of the same necklace, a problem made worse by the fact that looting, whether ancient or more recent, can alter the arrangement and remove some of the items originally present. The same consideration applies to fragments of gold sheet which may have sheathed organic materials, the original forms of which are unclear. Second, classifying beads separately provides a more direct (though still approximate) measure of the total amount of gold. A single bead contains significantly less gold than a necklace of many, so it would be somewhat misleading to classify both as one object if each was found in a separate grave. Worse, one might end up in a situation where three isolated beads in different graves from one site would be counted as more items than a necklace with 300 found in a single grave. Of course, objects like gold goblets, such as those from Karashamb, Trialeti, and Vanadzor, contain more gold than a single bead, so this is by no means an exact reflection of gold weight. Nevertheless, as beads and necklaces are far more common than larger single objects like goblets, this mode of counting and classification is the most reasonable approach. Likewise, fragments of gold sheet were generally treated as separate items in the database. In maps and graphs, scales were adjusted to such a way as to mitigate the effect of a small number of sites with hundreds of gold beads from swamping broader patterns. This decision is justifiable because taphonomy and archaeological luck, rather than actual ancient use patterns, are more likely at play in variations between sites with hundreds of gold objects. For example, the actual difference in gold use between a site with 800 objects and one with 400 objects is probably less significant than the difference between a site with 200 objects and one with 20. The factor-of-two difference in the former case is more likely a result of archaeological happenstance, while the factor-of-ten difference in the latter case may represent genuine differences in gold use. Order-of-magnitude quantitative variations were given greater weight in the interpretation, prompting the use of semi-log-scale plots for figures 2 and 5A in the main text.

Spatial precision of the gold findspots (**Supplementary Figs. S2-S5; Supplementary Table S1**) varied somewhat across the dataset. In some cases, the exact location of the site was known, while in many others, only the nearest modern settlement was known. In one case, the Zurtaketi kurgans, it was only possible to locate the finds to

a more general area. Nonetheless, even in this case, the spatial imprecision is small relative to the size of the region as a whole, and therefore it has little impact on the broader spatial patterning under discussion.

Locations of ore deposits are derived from two different sources. Maps in the Kalantarian volume (2007) cover modern day Eastern Turkey, Armenia, eastern Azerbaijan, with a few deposits in Southern Georgia, while the map published by Stöllner et al. (2014) covers modern Georgia and surrounding areas of the Greater Caucasus range, including southern Russia and eastern Azerbaijan. There is some overlap in coverage, and it is possible that in some areas, such as northern Armenia and southern Georgia, that the same deposit may be recorded twice. Together, they provide a reasonable picture of gold deposits in the Southern Caucasus, but there are important limitations to the data. First, as exact coordinates were not given for the deposits, coordinates were extracted by georeferencing the published maps and their spatial precision is not known. It is likely that these maps are compilations of older data derived from Soviet-era surveys with variable spatial precision. Nevertheless, the maps are sufficient for exploring regional-scale patterning. Second, the presence of a source of gold does not mean that it was exploited during the Bronze and Iron Age. Modern techniques permit the exploitation of gold deposits with grades in the parts per million, and heavy machinery permits mining of gold mineralizations at great depths. At the same time, work at the Sakdrisi gold mine suggests that even in the Early Bronze Age, metalworkers were capable of extracting gold from sub-surface hard-rock deposits (up to 31 m below ground) with fairly low gold concentrations (Stöllner et al. 2014). Indeed, there are hints of early gold mining at other deposits in the South Caucasus (Wolf et al. 2011), suggesting that the number of archaeologically-documented prehistoric gold mines is far less than the true number. The Kalantarian maps provide some indication of dates for different prospects, but because modern mining often obliterates earlier traces of exploitation, and prehistoric placer mining is extremely difficult to identify even in the best of circumstances, lack of mining evidence for a particular period does not prove that the deposit was not mined at this time. For these reasons, and because only one of the maps provided these chronological indications, all gold sources were treated equally in

spatial analysis. As a result, maps of gold sources should be considered *possible* sources for ancient exploitation, with no guarantee that they were mined in the Bronze Age.

Various spatial analyses were used to visualize the data and explore quantitative patterning. Circle-size plots were used to visualize both the numbers of sites with gold objects and the quantities of gold found at each site. The maximum size category was set well below the maximum number of objects found at a single site, to avoid overemphasizing exceptionally rich individual sites. Kernel density mapping provides an effective way of visualizing areas with high densities of gold objects, complementing the circle-size point maps, which illustrate both the numbers of sites and the quantities found at each. The search radius for the kernel density function was set at 30 km. This overlay of two data visualizations, as a density raster and as dot size plots, provides a clear picture of the ebb and flow of gold usage in the Caucasus and highlights the sharp contraction in the Middle Kura Zone and the northern Lesser Caucasus during the Late Bronze Age (**Fig. 3**).

Least-cost paths from the nearest gold deposit to each archaeological site were calculated with the ArcMap 10.8 Path Distance tool, using the Tobler hiking function, an anisotropic cost-weighting function, that assumes a maximum walking speed when travelling slightly downhill (Tobler 1993). The function assumes travel on pathways and yields costs in units of hours. Walking time was calculated *from* the nearest gold deposit to each site with gold objects, following the direction the gold would have theoretically travelled. Because of the Tobler function's subtle anisotropy, calculations of walking time from the findspots *to* the ore deposits would be slightly different, but most likely not to an extent that would have affected the overall interpretations. In addition, the model does not account for the effects on walking speed of groundcover, which may have differed in prehistory, or river crossings, making it a relative assessment of ease of access, rather than an exact measurement of travel time. All these considerations are unlikely to have a significant effect on the plots detailed in figures 4 and 5 of the main text.

Topographic inputs for the Tobler hiking function were calculated from a 3 arc-second digital elevation model (DEM) produced by the Shuttle Radar Tomography Mission (SRTM3), which has a horizontal resolution of approximately 90 m, a resolution

necessary to capture the many narrow gorges of the Caucasus region. To assess the impact of DEM resolution on the analysis, the same calculations were carried out on a lower-resolution 30 arc-second DEM (SRTM30). The smoothing introduced at lower resolutions yielded slightly shorter least cost travel times and, for a few sites, resulted in a different deposit being identified as the nearest, but the overall patterning of the quantities vs. walking time (**Fig. 5**) remained the same. This additional calculation provides confidence that minor approximations and simplifications in other aspects of the model would not appreciably change the overall picture of access.

The two key results of the least cost path analyses discussed in the main text are 1) that as a group, archaeological sites with gold in the Kura River valley are not significantly farther away from gold deposits than those in the Middle Araxes zone and 2) that distance from ore deposit does not effectively predict which areas experienced the most significant decreases in gold quantities in the Middle Bronze Age to Late Bronze Age transition.

One element of the argument is that fall off curves do not differ (**Fig. 5B**) dramatically between different periods, and that minor divergences (e.g. between the MBA and LBA/EIA curves at the 8-12 hour bin are most likely due to a small number of sites with significant gold quantities. **Supplementary Fig. S6** provides further graphical support for this contention, as it effectively visualizes the effect that a small number of gold-rich sites can have on quantities of objects plotted against distance from a deposit. The shift of one or two sites with a lot of gold between one bin is enough to cover the observed differences between fall off curves of different periods. As the nature of the data cautions against placing undue interpretive weight on features that may be the result of excavation patterns or lucky discoveries, it is clear that the data do *not* provide strong evidence for meaningful differences in the deposition of gold artifacts in relation to gold sources between different periods.

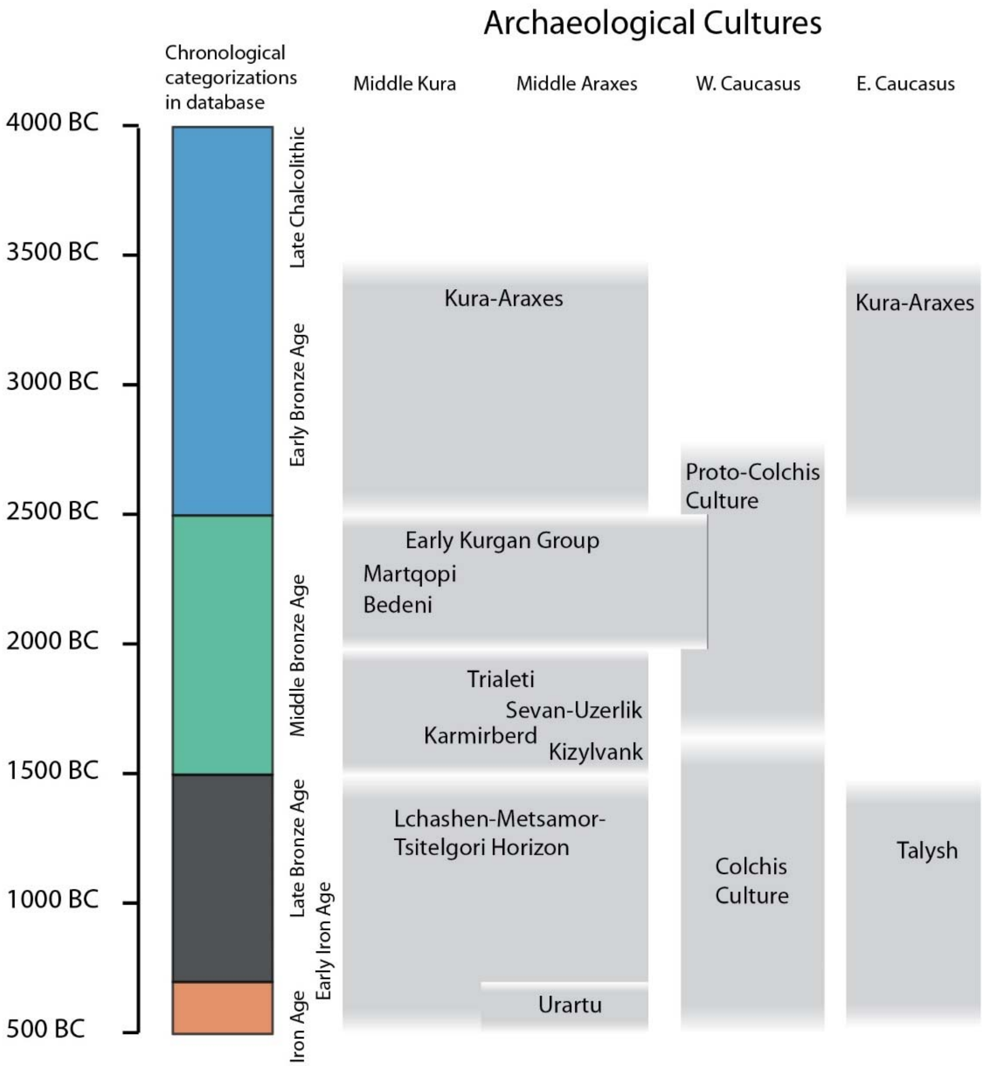

Supplementary Figure S1. Simplified schematic of archaeological cultures and chronologies in the South Caucasus, showing the chronological categorization in the database. Categorization colors correspond to those in Figure 5 in the main text. Chronological designations generally follow those in (Sagona 2018).

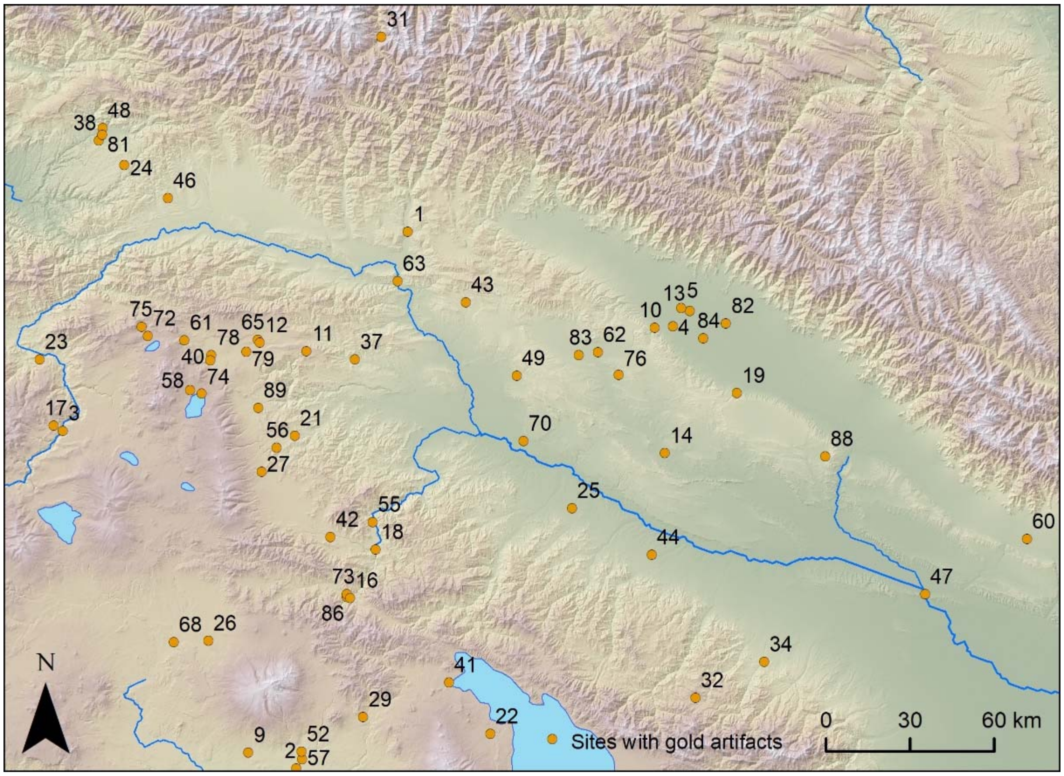

Supplementary Figure S2. Sites containing gold artifacts from the Middle Kura zone and immediately adjacent areas. Number labels correspond to those in Table S1.

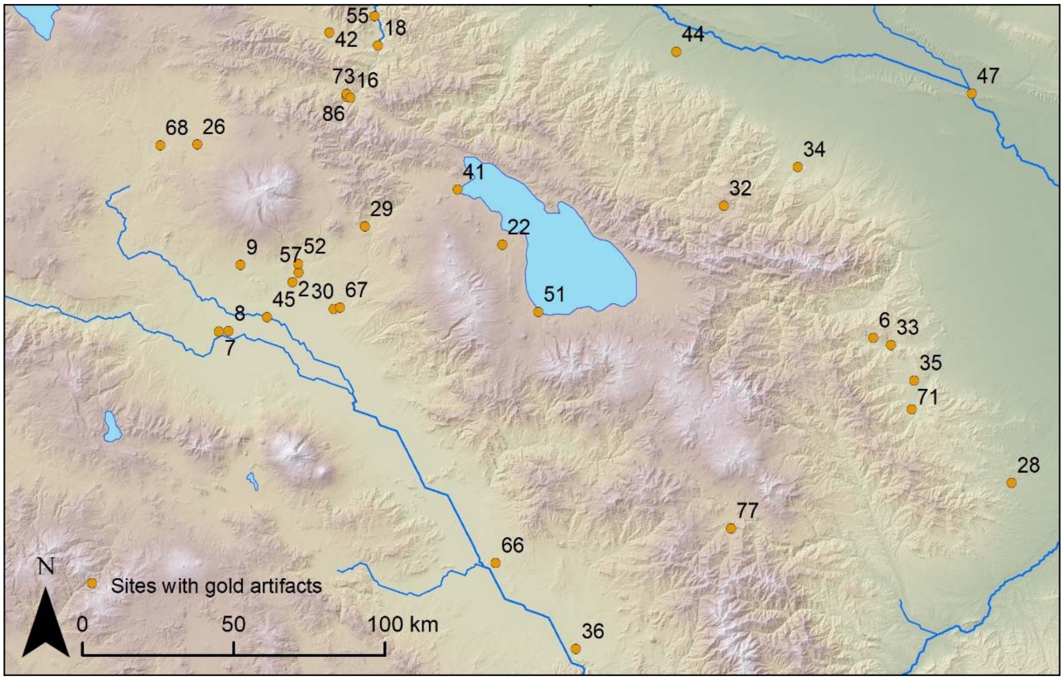

Supplementary Figure S3. Sites containing gold artifacts from the Middle Araxes zone and immediately adjacent areas. Number labels correspond to those in Table S1.

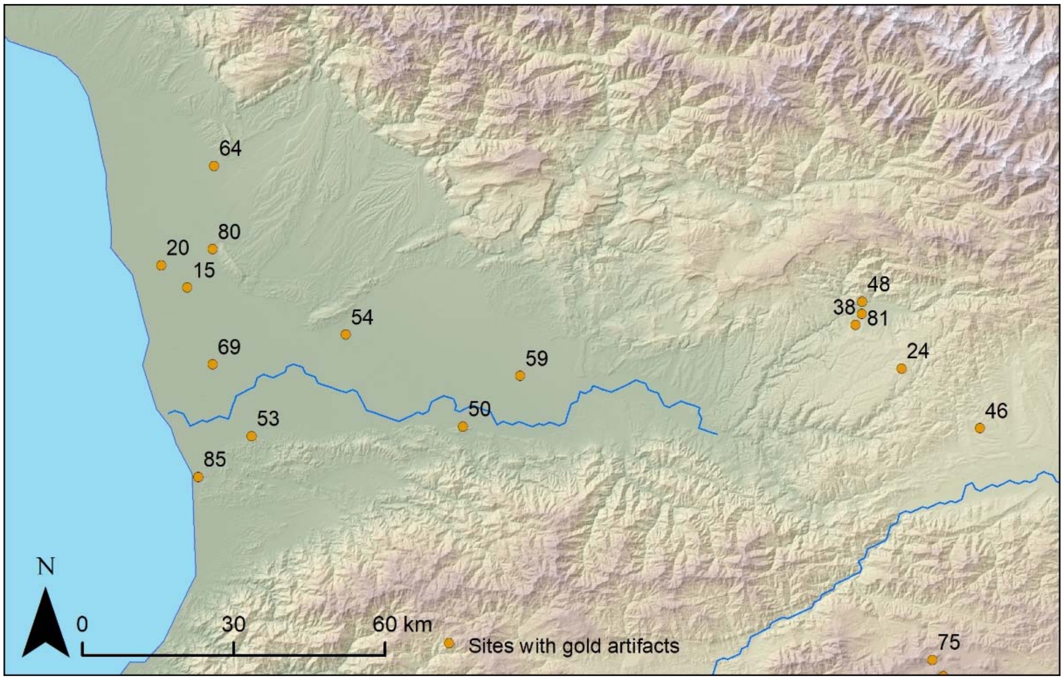

Supplementary Figure S4. Sites containing gold artifacts from the Western Caucasus zone. Number labels correspond to those in Table S1.

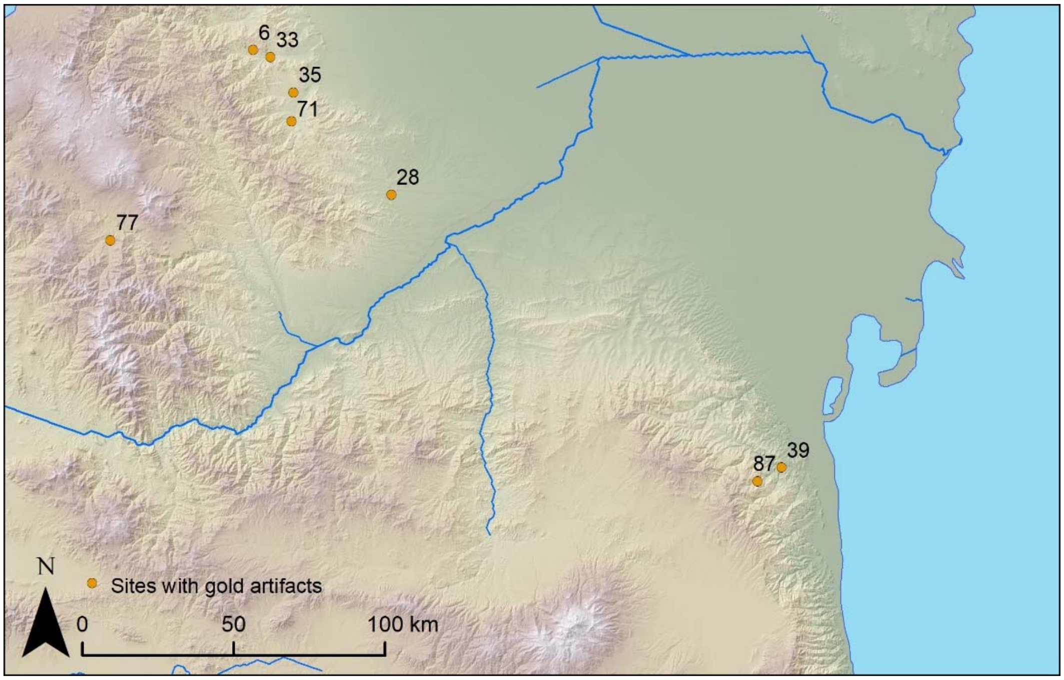

Supplementary Figure S5. Sites containing gold artifacts from the Eastern Caucasus zone and adjacent areas of the Middle Araxes zone. Number labels correspond to those in Table S1.

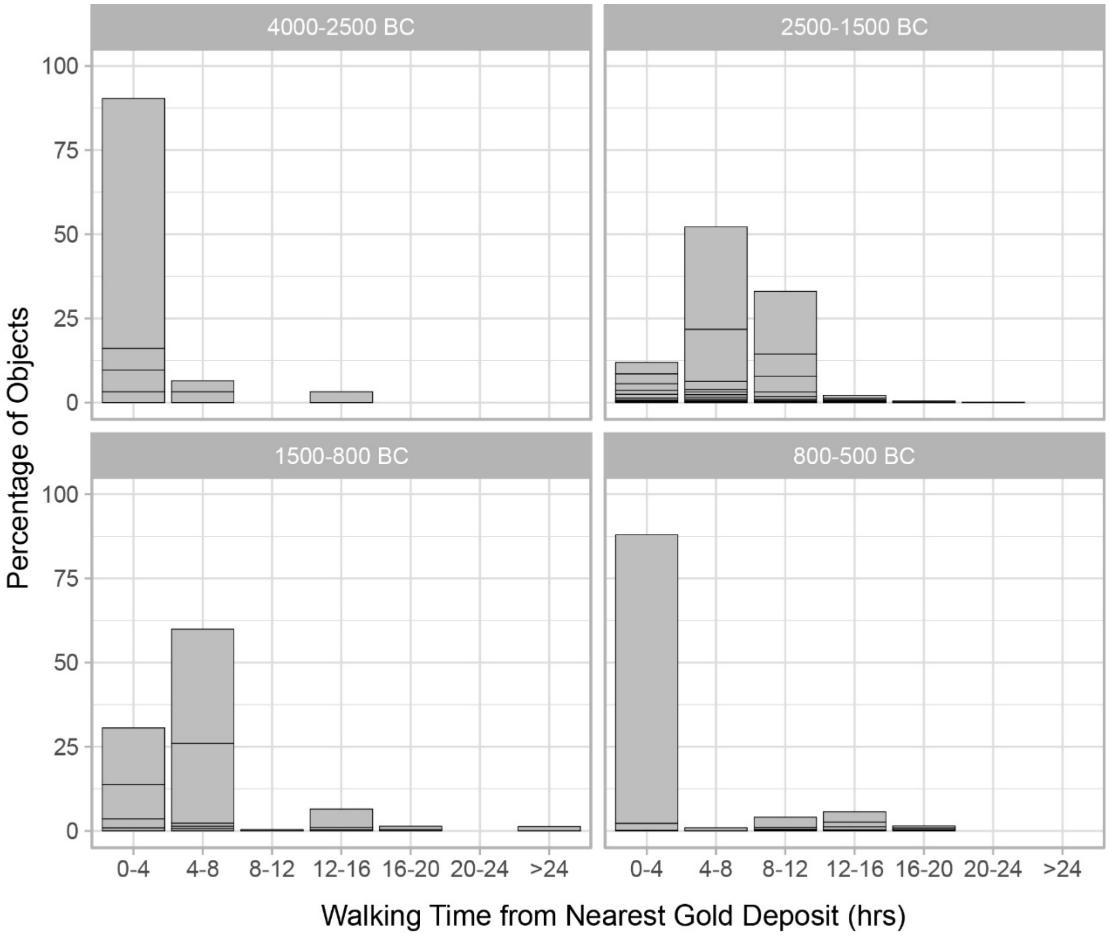

Supplementary Figure S6. Stacked bar charts showing the percentage of objects (for each period) binned against walking time from the nearest gold deposit. Divisions within each bar indicate objects from different sites. This figure is a companion to main text figure 5B, as it derives from the same data, but visualizes how the slight differences between the fall-off curves for different periods are impacted by a small number of sites with many gold objects.

Supplementary Table S1. Sites and Sources for the Database

Site Name	Map Label (fig. S2-S5)	Description	References
**Abanoskhevi**	1	Kurgan site north of Mtskheta near route to the Dariali pass over the Greater Caucasus range, dating to the 2nd half of the 3rd millennium BC.	(Ghambashidze et al. 2010)
**Aigeshat**	2	Site located east of Yerevan on the Ararat Plain. A late-3rd millennium BC gold item (a plaque) was found in one of the burials.	(Kalantarian 2007)
**Akhchia**	3	Group of kurgans located in the Kura gorge near the Georgian-Turkish border. Links were noted with the Middle Bronze Age Trialeti Culture with some local characteristics, suggesting dates in the 1st half of the 2nd millennium BC.	(Japaridze et al. 1985) (Japaridze et al. 1981)
**Anaga**	4	Kurgan located in the Alazani Valley, close to similarly dated Early Kurgan Culture kurgans Tsnori and Ananauri	(Ghambashidze et al. 2010)
**Ananauri**	5	Several very large kurgans, primarily belonging to the Early Kurgan culture. They date to the mid-late 3rd millennium BC.	(Lordkipanidze 2016) (Makharadze et al. 2016) (Makharadze and Murvanidze 2014) (Ghambashidze et al. 2010)
**Archadzor**	6	Burials dating to the Late Bronze-Early Iron Age excavated in the late 19th century in the lesser Caucasus foothills of Nagorno-Karabakh.	(Rösler 1894) (Dzhafarov 1984) (Rösler 1896a)
**Argishtikhinili**	7	An Urartian fortress site located in the Ararat Valley. Substantial excavations took place here during the Soviet era.	(Martirosyan 1974) (Kalantarian 2007)
**Armavir**	8	Sister-site to Argishtikhinili (the neighboring Urartian-period center). Best known as a Classical era site, the two gold objects in the database date to the 7th-6th century BC.	(Kalantarian 2007)
**Aruch**	9	Middle Bronze Age mortuary site at the edge of the Ararat Plain dating to the 1st half of the 2nd millennium BC; gold objects are reported from two burials.	(Kalantarian 2007)

**Bakurtsikhe**	10	Kurgan in the Alazani Valley dating to the Early Kurgan Cultures (2nd half of 3rd millennium BC)	(Ghambashidze et al. 2010)
**Bedeni**	11	Type site for the Bedeni Culture, is one of several cultures delineated in the mid-late 3rd millennium BC "Early Kurgan Cultures" group, which is variously assigned to the EBA or MBA. Numerous kurgans were excavated at the site, including cart burials.	(Ghambashidze et al. 2010) (Gobejishvili 1981)
**Beshtasheni**	12	Originally excavated by Kuftin just before the Second World War. A small number of gold artifacts at the site date to the Late Bronze Age, but the site has Early Bronze Age remains as well.	(Kuftin 1941) (Hauptmann et al. 2010)
**Chinchriani Gora**	13	A kurgan site in the Alazani Valley	(Makharadze 2018)
**Dalis Mta**	14	Kurgan site in Kakheti located close to the modern Azerbaijani-Georgian border. Kurgan #4, which contains gold objects, dates to the second half of the 3rd millennium BC	(Ghambashidze et al. 2010)
**Dghvaba**	15	A collective grave site in the Colchian lowlands dating to the Iron Age (roughly 8th-6th c. BC).	Personal observation, Poti Museum of Colchian Culture, 2013-2014.
**Dimats**	16	Site in the Vanadzor area in northern Armenia. Context of the Late Bronze gold finds (e.g. mortuary vs. settlement or even chance find) is unclear, Iron Age object derives from a grave.	(Kalantarian 2007)
**Dumeila**	17	Located on the plateau on the left bank of the Kura River near the Georgian-Turkish border. Links were noted with the Middle Bronze Age Trialeti Culture with some local variants, suggesting dates in the 1st half of the 2nd millennium BC.	(Japaridze et al. 1985)
**Dzora**	18	Chance find near the Dzora/Dzoraget Hydroelectric station.	(Kushnareva 1997)
**Enamta**	19	Kurgan site located at the edge of the Alazani Valley near the Georgian-Armenian border.	(Ghambashidze et al. 2010)
**Ergeta**	20	Consists of several collective grave sites in the Colchian lowlands near the modern	(Mikeladze et al. 1985)

		village of Ergeta. (Ergeta I, Ergeta II, etc.) Large quantities of metal objects and other craft items are found deposited in these graves. The majority likely date to the 8th-6th centuries BC.	(Mikeladze et al. 1984) (Papuashvili 2011)
**Gantiadi**	21	Cemetery site in the Mashavera gorge, containing many graves from the 2nd and 1st millennium BC. The gold objects recorded in the database belong the 8th-7th c. BC.	(Hauptmann et al. 2010) (Kakhiani et al. 1985)
**Gavar "Mrtbi Dzor"**	22	Late Bronze-Early Iron Age site near Lake Sevan. Gold finial on an agate bead in the database comes from a 13th-12th c. BC grave.	(Kalantarian 2007)
**Gokhebi**	23	Mortuary in the Samtskhe region, Gold-bearing kurgan was dated to the early 2nd millennium BC (Middle Bronze Age).	(Hauptmann et al. 2010) (Gambashidze and Kvizhinadze 1981)
**Goradziri**	24	Goradziri is a site located in the Sachkhere region of Imereti, western Georgia. Excavations uncovered several kurgans and other burials. Gold was found in a 7th/6th century burial.	(Gogadze and Davlianidze 1981)
**Hasansu**	25	Hasansu is a kurgan burial site in western Azerbaijan, with Late Kura-Araxes period remains, roughly the mid-3rd millennium BC. While this falls at the boundary in the chronological characterizations used in the present work, the association with the Kura-Araxes culture favors a grouping with the Late-Chalcolithic to Early Bronze Age assemblages.	(Courcier et al. 2017) (Jansen et al. 2014)
**Horom**	26	Horom is a major Late Bronze and Early Iron Age fortified site in the Shirak plain of northern Armenia. Gold artifacts come from 9th-8th c. BC burials at the site.	(Kalantarian 2007) (Badaljan et al. 1992)
**Irganchai**	27	Kurgans located near Irganchai village on the Georgian-Armenian border.	(Hauptmann et al. 2010)
**Karabulak**	28	Series of kurgans excavated during the late 19th century at the edge of the lesser Caucasus foothills north of the Araxes River. Composite objects with gold were excavated from Kurgan #5 (alternatively numbered burial #91 in the publication).	(Ivanovskiy 1911)

**Karashamb**	29	Major kurgan site attributed to the end of the 3rd millennium BC, most well-known for the silver Karashamb goblet discovered in 1987, which echoes very closely a similar silver goblet found by Kuftin at Trialeti decades earlier. Large quantities of gold were also found in this kurgan.	(Kalantarian 2007) (Oganesian 1992) (Rubinson 2013)
**Karmir Blur**	30	Urartian fortress site located on the outskirts of modern Yerevan, designed to control the Ararat plain after the Urartian conquest of the region. Remains date primarily to the 7th and early 6th c. BC, but most of the gold finds are attributed to slightly earlier periods (8th/7th c. BC) The site is one of the few where gold has been found in a non-mortuary context.	(Kalantarian 2007) (Piotrovskiy 1955) (Piotrovskiy 1952) (Piotrovskiy 1950)
**Kazbegi**	31	The approximate location of several chance finds—some said to be from a hoard, found in and around the town of Kazbegi (modern Stepantsminda), on the main route through the central Greater Caucasus range.	(Kuftin 1941) (Gamkrelidze 2012) (Menabde and Davlianidze 1968)
**Khachbulag**	32	Also referred to as Hacibulak. Gold was found here in a grave with Kura-Araxes pottery	(Narimanov 2004) (Courcier 2014)
**Khachenaget**	33	Kurgans excavated in the late 19th century in Nagorno-Karabakh. In some sources these are referred to as Early Bronze Age, but based on the strong similarity of the goldwork to Trialeti Kurgan L, dated to the mid-3rd millennium and associated with the Early Kurgan Cultures, in the analyses these remains are assigned to the 2500-1500 chronological categorization.	(Rösler 1899) (Dzhafarov 1984)
**Khanlar**	34	Site located south of the modern city of Ganja, Azerbaijan. Of 300 graves excavated in this area, only one grave contained gold—a single bead from kurgan #2.	(Dzhafarov 1984) (Gummel 1949)
**Khodzhaly**	35	Kurgan burials excavated in the late 19th and first half of the 20th century in Nagorno-Karabakh. The burials are generally dated to the Late Bronze and Early Iron Age, but where in the range of 1500-500 BC is not always clear. Most burials were categorized the 1500-800 BC	(Dzhafarov 1984) (Rösler 1896b) (Rösler 1898)

		chronological category, except Kurgans #11 and #14. Kurgan #11 contains a bead with possible cuneiform associated with Assyrian king Adad-Nirari, one of several with that name in the late 2nd and early 1st millennium BC. As some of those who argue for Adad-Nirari I (late 2nd mill. BC) suggest it arrived in the 8th century BC, and several others favor other Adad-Niraris in the 1st millennium BC, Kurgan #11, and Kurgan #14, which is very similar, are placed in the 800-500 BC category.	
**Kizylvank**	36	Cemetery site in Nakhchivan, located near the monastery of Kizylvank. The type site for the Middle Bronze Age Kizilvank Culture, but it is possible that LBA materials are present. A gold ring (an earring?) was excavated at the site in the early 20th century.	(Dzhafarov 1984) (Spitsyn 1909) (Kushnareva 1997)
**Kobal**	37	A site in the Algeti gorge (Kvemo Kartli) excavated in association with the construction of a reservoir. A grave in the Kobal cemetery (#10), dated to the late Middle Bronze Age, contains gold.	(Tushishvili 1981)
**Koreti**	38	A burial site (kurgan), part of a complex of sites near Sachkhere, western Georgia, which is attributed to a transitional EBA-MBA period (mid-3rd millennium BC).	(Ghambashidze et al. 2010)
**Kraveladi II**	39	Kraveladi II is a Late Bronze-Early Iron age cemetery cite in southeast Azerbaijan. The area was originally investigated by Jacques de Morgan in the late 19th century, but the gold bead recorded in the database comes from a new French-Azerbaijani project.	(Casanova et al. 2016)
**Kushchi**	40	Also, called Trialeti Kurgan XXXIII, this kurgan is somewhat separated from the main area of Trialeti kurgans, so it is listed as a separate site. It dates to the Middle Bronze Age (1st half of the 2nd millennium BC).	(Kuftin 1941) (Zhorzhikashvili and Gogadze 1974)
**Lchashen**	41	Major type site for the Late Bronze Age and Early Iron Age sequence, defining the Lchashen-Metsamor (also termed the Lchashen-Tsitelgori) horizon. At this site, located on the shores of Lake Sevan,	(Kalantarian 2007) (Mnatsakanyan 1961)

		elaborate cart burials last into the later 2nd millennium BC.	
**Lori Berd**	42	Site dating to the Middle Bronze Age, Late Bronze, and Iron Age periods. One of the northernmost sites with significant quantities of gold in the Late Bronze Age. Gold primarily comes from graves at the site.	(Kalantarian 2007)
**Martqopi**	43	Type site for the Martqopi Culture, consisting of kurgan burials. The Martqopi Culture is one of several delineated in the mid-late 3rd millennium BC Early Kurgan Cultures, which are variously assigned to the EBA or MBA.	(Ghambashidze et al. 2010)
**Mentesh Tepe**	44	A Neolithic settlement that was re-used for a kurgan-like burial in the Early Kurgan period (Martqopi Culture). The burial was radiocarbon dated to the 25th. c. BC.	(Pecqueur et al. 2017)
**Metsamor**	45	A multi-period settlement site with surrounding cemeteries in the Ararat Valley. Gold artifacts primarily from graves at the site.	(Kalantarian 2007)<br>(Jakubiak et al. 2017)
**Mikelaant Verkhvebi**	46	A reference to unspecified gold objects is reported in this cemetery site in Shilda Kartli dating to the “end of the Middle Bronze Age and the transitional period from Middle to Late Bronze Age”	(Ramishvili 1981)
**Mingeçevir**	47	This complex of sites in and around the territory flooded by the Mingeçevir reservoir includes sites of a range of different periods. Often referred to in the Russian literature as Mingechaur. The modest amounts of gold in the database coming from these sites dates to the Early Bronze Age (Kura-Araxes culture), the Late Bronze-Early Iron Age, and the 7th-5th c. BC.	(Aslanov et al. 1966)<br>(Aslanov et al. 1959)<br>(Courcier 2014)<br>(Narimanov 2004)
**Modinakhe**	48	Site of a medieval castle, some mid-3rd millennium BC graves excavated here part of a complex of sites near Sachkhere, western Georgia, which is attributed to a transitional EBA-MBA period.	(Ghambashidze et al. 2010)
**Mravaltskali**	49	A Middle Bronze Age burial site in the Kura Valley, close to the Georgia-Azerbaijan border.	(Hauptmann et al. 2010)

**Mtisdziri**	50	A settlements site in Colchis. It is unclear whether the 6th century BC gold earrings in the database come from a settlement context or a nearby grave.	(Menabde and Davlianidze 1968) (Apakidze 2005)
**Nerkin Getashen**	51	Gold artifacts from this site, on the edge of Late Sevan, come from a grave dated to the end of the 3rd millennium BC.	(Kalantarian 2007)
**Nerkin Naver**	52	A kurgan burial site of the late 3rd millennium BC located in the Ararat.	(Kalantarian 2007) (Simonyan and Manaseryan 2013)
**Nigvziani**	53	A mortuary site consisting of collective graves and associated cult platforms, dated to the 7th-6th c. BC.	(Mikeladze 1985) (Mikeladze and Baramidze 1977)
**Nosiri**	54	A hoard from the village of Nosiri is mentioned by Kuftin, but precise context is not given. Kuftin dates it generally to the first half of the 1st millennium BC, but when viewed in the context of more recent finds, placement in the 800-500 BC chronological category seems most justifiable. Kuftin himself notes similarities with material from Karmir Blur, an Urartian site founded in the 7th c. BC.	(Kuftin 1941)
**Odzun**	55	Finds of unclear context, including chance finds, near modern Odzun, northern Armenia, dating to the late 3rd and. Also referred to as Uzunlar	(Kalantarian 2007)
**Orozmani**	56	Kurgan site in Kvemo Kartli, Georgia, dating to the Middle Bronze Age (the first half of the 2nd millennium BC).	(Hauptmann et al. 2010)
**Oshakan**	57	Urartian-period fortress site in the Ararat Plain. The specific context of the gold artifacts, whether excavated from the fortress or from graves nearby, is unclear in the Kalantarian volume.	(Kalantarian 2007)
**Paravani**	58	Early 3rd millennium BC kurgan excavated in 2003-2005 near the village of Akhali Khugumlo near the shores of Lake Paravani.	(Ghambashidze et al. 2010) (Kvavadze and Kakhiani 2010)
**Partskhanakanevi**	59	19th century finds reported on by Kuftin in the collection of the state museum. Kuftin assigns the finds to the same period as the Nosiri gold finds. Unlike the Nosiri finds,	(Kuftin 1941)

		which are clearly referred to as a hoard, it is unclear whether the Partskhanakanevi materials come from a hoard or burial.	
**Qarajemirli**	60	A kurgan cemetery site in the Oğuz region of Azerbaijan, dating to the Early Kurgan Period (Martqopi-Bedeni cultures)	(Courcier et al. 2016)
**Sabit Akhcha**	61	A group of kurgans somewhat separated from the main area of Trialeti kurgans, so it is listed as a separate site. It dates to the Middle Bronze Age (1st half of the 2nd millennium BC). Trialeti Kurgan XXIX belongs to this group.	(Japaridze 1960) (Kuftin 1941) (Zhorzhikashvili and Gogadze 1974)
**Saduga**	62	A kurgan (#4) excavated near the Iori river in Kakheti, Georgian, dating to the Middle Bronze Age (Trialeti Culture). While most of the artifacts, including the gold object, are solidly Middle Bronze Age, there is one vessel that might be characteristic of the Middle-Late Bronze Age transition.	(Pitskhelauri 1984)
**Samtavro**	63	A site best known for its Late Bronze-Early Iron Age settlement and cemeteries, as well as Classical to Late Antique cemeteries, a smaller number of Middle Bronze Age graves are known from the site. The gold recorded in the database comes from an MBA grave (#243).	(Kalandadze 1980)
**Samurzakan**	64	A hoard mentioned by Kuftin as similar to the Nosiri hoard. Kuftin dates the Samurzakan hoard to the Late Bronze Age, but the mention of gold and silver objects suggests, in light of more recently excavated materials, that a date somewhere in the range of 800-500 BC is more likely.	(Kuftin 1941)
**Sapar Kharaba**	65	Late Bronze Age cemetery on the Tsalka Plateau, not far from the major Trialeti culture Middle Bronze Age kurgans. Given the number of graves excavated at this site, the paucity of gold is a remarkable contrast to burials of the preceding Middle Bronze Age.	(Narimanishvili 2010) (Hauptmann et al. 2010)
**Şahtaxtı**	66	Cemetery site in Nakhchivan, Azerbaijan. Also sometimes written as Shakhtakhty or Shakh-Takhty. Dating of the site (and the gold plaque found in one of the graves) to either the (late) Middle or (early) Late	(Kushnareva 1997) (Aslanov et al. 1966) (Dzhafarov 1984)

		Bronze Age is challenging, as comments are vague, imprecise, or contradictory.	(Kushnareva 1993) (Alekperov 1937) (Kuftin 1941)
**Shengavit**	67	Early Bronze Age site located on the edge of modern Yerevan in the Ararat Plain	(Kalantarian 2007)
**Shirakavan**	68	Late Bronze Age (14th-13th c. BC) graves were excavated at Shirakavan, located in northwestern Armenia on the Shirak Plain.	(Kalantarian 2007)
**Simagre**	69	Settlement site in the Colchian lowlands near the mouth of the river Phasis, with remains in the pre-Classical and Classical periods.	
**Soyuq Bulaq**	70	A series of Late Chalcolithic (1st half of 4th millennium BC) kurgans were excavated at this site in Azerbaijan, close to the Georgian border and adjacent to the Kura River.	(Courcier et al. 2017) (Lyonnet et al. 2008)
**Stepanakert**	71	Located in Nagorno-Karabakh, the Stepanakert kurgans were excavated in the mid-20th century. Publications at the time attribute them to the "Early Bronze Age," but reexamination of associated finds suggest that they belong to the Early Kurgan cultures group (mid-late 3rd millennium BC; variously designated at Early or Middle Bronze age in different chronologies) rather than the Kura-Araxes Culture.	(Gummel 1948)
**Tabatsquri**	72	A kurgan excavated near Lake Tabatsquri in Javakheti, Georgia.	(Japaridze 1960)
**Tagavoranist**	73	A site with Late Bronze-Early Iron Age materials close to Vanadzor, northern Armenia.	(Kalantarian 2007)
**Taparavani**	74	A group of kurgans separated from the main area of Trialeti kurgans, so it is listed as a separate site. The gold-containing kurgan in the database was excavated by Kuftin in the mid-20th century and is also referred to as Trialeti Kurgan XL.	(Kuftin 1941) (Zhorzhikashvili and Gogadze 1974)
**Tavkvetili**	75	A kurgan site in southern Georgia dating to the first half of the 2nd millennium BC.	(Hauptmann et al. 2010)
**Tetri Kvebi**	76	Mid-3rd millennium kurgan site in the Iori valley (Kakheti, eastern Georgia).	(Ghambashidze et al. 2010)

**Tolors**	77	Kurgan dating to the 12th-11th c. BC in Syunik, southern Armenia.	(Kalantarian 2007)
**Top Kar**	78	A group of kurgans excavated by Kuftin in the mid-20th century, but somewhat separated from the main area of Trialeti kurgans, so it is listed as a separate site. The gold containing Top Kar kurgans in the database correspond to Trialeti Kurgan XXXVI and Trialeti Kurgan XLV.	(Kuftin 1941) (Zhorzhikashvili and Gogadze 1974)
**Trialeti**	79	A group of kurgans burials on the Tsalka plateau (modern Georgia) which are typically grouped together and which serve as the type site for the Middle Bronze Age Trialeti Culture.	(Kuftin 1941) (Zhorzhikashvili and Gogadze 1974)
**Tsaishi**	80	Mortuary site containing two collective graves dating to the 8th-7th century BC. Aside from gold artifacts, the collective graves contain a large and diverse assemblage of metal and non-metal grave goods.	(Papuashvili 2012)
**Tsartsis Gora**	81	A burial site (kurgan), part of a complex of sites near Sachkhere, western Georgia, which is attributed to a transitional EBA-MBA period (mid-3rd millennium BC).	(Kuftin 1949) (Ghambashidze et al. 2010)
**Tsitelgori**	82	Site containing Late Bronze Age burials in the Alazani Valley. Though this site is one of the few Late Bronze Age site in the Middle Kura zone with gold artifacts, the overall quantity—even in this otherwise wealthy grave, contrasts to the Alazani kurgans of the preceding periods. Also called Ulyanovka after a nearby settlement.	(Dedabrishvili 1979) (Abramishvili and Abramishvili 2008)
**Tsitsmatiani**	83	Kurgan near the Iori river, eastern Georgia. No grave objects were found in the central burial, but an MBA gold bead was found on the neck of a skeleton that was interpreted as a looter trapped by collapse.	(Pitskhelauri 1984)
**Tsnori**	84	A group of several very large gold-rich kurgans located in the Alazani Valley belonging to the Early Kurgan Cultures group. Kurgan #1 is 11 m tall and measures 168 m × 136 m, while Kurgan #2 measures 80 m × 90 m with a height of 2.5 m.	(Dedabrishvili 1979) (Ghambashidze et al. 2010)
**Ureki**	85	Ureki is an early 1st millennium BC cemetery site located near the Black Sea coast. It includes both individual and	(Mikeladze 1985)

		collective graves, some of which contain very large quantities of grave goods, including gold.	
**Vanadzor**	86	Burials of the late 3rd millennium BC contain gold artifacts, mostly notably an exceptional gold cup with lion decoration.	(Kalantarian 2007)
**Veri**	87	Cemetery site with Late Bronze and Early Iron Age graves in southeastern Azerbaijan that was excavated by Jacques de Morgan in the late 19th century.	(de Morgan 1896)
**Zilicha**	88	An Early Kurgan site located in Kakheti, near the Georgian-Armenian border.	(Ghambashidze et al. 2010)
**Zurtaketi**	89	A number of Middle Bronze Age kurgans were excavated in the region of Zurtaketi. Exact kurgan locations are not reported, but the approximate location is known.	(Japaridze 1969)

Supplementary Dataset S1 (separate file). Database of gold objects used in the study. The file contains four sheets. The "Object List" sheet provides information about each gold object in the database and a reference. The "Site Calculations" sheet details the number of objects found at each site, as well as the least cost walking time and distance from the nearest gold deposit. The "Regional Calculations" sheet records the numbers of objects for each region in different periods (raw data for main text figure 2). The "Least Cost Calculations" sheet reports numbers of objects against walking times from the nearest deposits (raw data for main text figure 5).

Supplementary Dataset S2 (separate file). Geodatabase (prepared in ArcMap 10.8) containing spatial data on archaeological sites with gold objects, gold deposit locations, least cost paths used for the analyses. Also included in the .zip file are the path distance and backlink rasters used to generate the least cost paths.

Supplementary Dataset S3 (separate file). R code and associated data formatted as .csv files used to produce Figs. 2, 5, and Supplementary Fig. S6.

References

Abramishvili, R. and Abramishvili, M., 2008, Late Bronze Age Barrows at Tsitelgori, in *Archaeology in the Southern Caucasus: Perspectives from Georgia* (eds. A. Sagona and M. Abramishvili), Peeters, Leuven, 351-363.

Akhvlediani, N., 2005, Problems of the chronology of Late Bronze Age and Early Iron Age sites in eastern Georgia (Kvemo Sasireti hoard), *Ancient Civilizations from Scythia to Siberia,* **11**, 257-295.

Alekperov, A., 1937, Krashenaya keramika Nakhichevanskogo Kraya a Vanskoe Tsarstvo, *Sovetskaya Arkeologiya,* **4**, 249-263.

Apakidze, J., 2005, Towards the study of Late Bronze Age and Early Iron Age settlements and settlement systems of the Colchian Culture in Western Georgia, *Archäologische Mitteilungen aus Iran und Turan,* **37**, 175-187.

Aslanov, G. M., Golubkina, T. I., and Sadykhzade, 1966, *Katalog zolotykh n serebryanykh predmetov iz arkheologicheskikh raskopok Azerbaydzhana,* AN Azerbaydzhanaskoy SSR, Baku.

Aslanov, G. M., Vaidov, R. P., and Ione, G. I., 1959, *Drevniy Mingechaur,* Akademii Nauk Azerbaydzhanskoy SSR, Baku.

Badaljan, R. S., Edens, C., Kohl, P. L., and Tonikjan, A., 1992, Archaeological investigations at Horom in the Shirak Plain of northwestern Armenia, *Iran,* **30**, 31-48.

Bukhrashvili, P., Blocher, F., Tskvitinidze, Z., and Davitashvili, S., 2019, Ausgrabungen in Nazarlebi, Kachetien (Georgien) 2017 und 2018, *Mitteilingen der Deutschen Orient-Gesellschaft zu Berlin* **151**, 271-294.

Casanova, M., Lorre, C., Alekberov, A., Fahimi, H., Sauvage, M., Haze, M., and Vella, M.-A., 2016, The necropolises of Azerbaijan during the Bronze and Iron Ages in the Lenkoran and the Lerik areas (NABIALLA Project, Azerbaijan): Preliminary report of the 2012–2013 campaigns, *Quaternary International,* **395**, 194-207.

Courcier, A., 2014, Ancient metallurgy in the Caucasus from the sixth to the third Millennium BCE, in *Archaeometallurgy in Global Perspective* (eds. B. W. Roberts and C. P. Thornton), Springer, New York, 579-664.

Courcier, A., Jalilov, B., Aliyev, I., Guliyev, F., Jansen, M., Lyonnet, B., Mukhtarov, N., and Museibli, N., 2016, The ancient metallurgy in Azerbaijan from the end of the Neolithic to the Early Bronze Age (6th to 3rd millennium BCE): An overview in the light of new discoveries and recent archaeometallurgical research, in *From bright ores to shiny metals: Festschrift for Andreas Hauptman on the occasion of 40 years research in archaeometallurgy and archaeometry* (eds. G. Körlin, M. Prange, T. Stöllner, and Ü. Yalçin), Deutsches Bergbau-Museum, Bochum, 26-36.

Courcier, A., Ragimova, M., Museibli, N., and Bakhtiyar, J., 2017, Metallurgical developments in Azerbaijan from the Neolithic to the Early Bronze Age, in *At the Northern Frontier of Near Eastern Archaeology: Recent Research on Caucasia and Anatolia in the Bronze Age* (eds. E. Rova and M. Tonussi), Brepols, Turnhout, Belgium, 525-541.

Danti, M. D., 2014, The Hasanlu (Iran) Gold Bowl in context: all that glitters…, *Antiquity,* **88**, 791-804.

de Morgan, J., 1896, *Mission Scientifique en Perse, Vol. IV,* Leroux, Paris.

Dedabrishvili, S., 1979, *Kurgany Alazanskoj Doliny,* Metsniereba, Tbilisi.

Dzhafarov, G. F., 1984, *Svyazi Azerbaydzhana co Stranami Peredney Azii v Epokhu Pozdney Bronzy n Rannego Zheleza,* Elm, Baku.
Flad, R., 2002, Ritual or structure? Analysis of burial elaboration at Dadianzi, Inner Mongolia, *Journal of East Asian Archaeology,* **3**, 23-51.
Gambashidze, P. and Kvizhinadze, K., 1981, Raboty Meskhet-Dzhavakhetskoy Ekspeditsii, in *Polevye Arkheologicheskiye Issledovaniya v. 1978 godu* (eds. O. Lordkipanidze, T. MIkeladze, and I. Dzhalagania), Metsniereba, Tbilisi, 57-64.
Gamkrelidze, G., 2012, *Researches in Iberia-Colchology (History and Archaeology of Ancient Georgia),* Georgian National Museum, Tbilisi.
Geselowitz, M. N., 1993, Archaeology and the social study of technological innovation, *Science, Technology, & Human Values,* **18**, 231-246.
Ghambashidze, I., Mindiashvili, G., and Gogoch'uri, 2010, *Udzvelesi metalurgia da samto sakme sakartveloshi dzv. ts'. VI-III atasts'leulebshi,* Mts'ignobari, Tbilisi.
Gobejishvili, G., 1981, *Bedenis gorasamarxebis k'ult'ura,* Metsniereba, Tbilisi.
Gogadze, E. and Davlianidze, T., 1981, *Goradziri,* Metsniereba, Tbilisi.
Gummel, Y. I., 1948, Hekotorye pamyatniki rannebronzovoy epokhi Azerbaydzhana, *Kratkie Soobshchenie Instituta Istorii Material'noy Kul'tury (KSIIMK),* **20**, 15-28.
Gummel, Y. I., 1949, Kurgan №2 bliz Khanlara, *Kratkie Soobshchenie Instituta Istorii Material'noy Kul'tury (KSIIMK),* **24**, 55-58.
Hauptmann, A., Bendall, C., Brey, G., Japaridze, I., Ghambashidze, I., Klein, S., Prange, M., and Stöllner, T., 2010, Gold in Georgien. Analytische Untersuchungen an Goldartefakten und an Naturgold aus dem Kaucasus und dem Transkaukasus, in *Von Majkop bis Trialeti. Gewinnung under Verbreiung von Metallen und Obsidian in Kaukasien im 4-2 Jt. v. Chr.* (eds. S. Hansen, A. Hauptmann, I. Motzenbäcker, and E. Pernicka), Rudolf Habelt GmbH, Bonn.
Ivanovskiy, A. A., 1911, *Po Zakavkaz'yu. Arkheologicheskiya Nabludeniya i Isledovaniya 1893,1894, i 1896 gg.,* Materialy po Arkheologii Kavkaza*,* A. H. Mamontova, Moskva.
Jakubiak, K., Iskra, M., Piliposyan, A., and Zakyan, A., 2017, Preliminary report on the 2016 season in Metsamor (Armenia), *Polish Archaeology in the Mediterranean,* **26**, 561-573.
Jansen, M., Stöllner, T., and Courcier, A., 2014, Appendix: Recent results from laboratory work on the gold of Sakdrisi, in *Metalle der Macht – Frühes Gold und Silber* (eds. H. Meller, R. Risch, and E. Pernicka), Landesamt für Denkmalpflege und Archäologie Sachsen-Anhalt, Halle, 105-108.
Japaridze, O., 1960, *Arkeologiuri Gatkhrebi Trialetshi 1957-1958 Ts'. (Archaeological Excavations in Trialeti, 1957-1958),* Tbilisi State University, Tbilisi.
Japaridze, O., 1969, *Arkeologiuri Gatkhrebi Trialetshi: Kartveli T'omebis Istoriisatvis Dzv. Ts'. II Atasts'leulshi (Archaeological Excavations in Trialeti: On the History of Georgian Tribes in the Second Millennium BC),* Sabch'ota Sakartvelo, Tbilisi.
Japaridze, O., Avalishvili, G. B., and Tsereteli, A. T., 1985, *Pamyathiki Meskheti Epokhi Sredney Bronzy,* Tbilisi University Press, Tbilisi.
Japaridze, O., I., K., Avalishvili, G., and Tsereteli, A., 1981, *Meskhet-Javakhetis Arkeologiuri Eksp'editsiis Mushaobis Shedegebi (1970-1977 tsts.),* Metsniereba, Tbilisi.

Kacharava, D. D., 2005, Recent finds at Vani, *Dialogues d'Histoire Ancienne,* **Supplément 1**, 291-309.
Kacharava, D. D. and Kvirkvelia, G. T., 2008, *Wine, worship, and sacrifice: the golden graves of ancient Vani,* Institute for the Study of the Ancient World, in association with Princeton University Press, Princeton, NJ.
Kakhiani, K. K., Iremashvili, S. A., Iordanashvili, Z. V., and Tskvitinidze, Z. R., 1985, Arkheologicheskaya Ekspeditsiya Mashaverskogo Ushchel'ya, in *Polevye Arkheologicheskiye Issledovaniya v. 1982 godu* (eds. O. D. Lordkipanidze, T. K. Mikeladse, and I. L. Dzhalagania), Metsniereba, Tbilisi, 29-31.
Kalandadze, A. N., 1980, *Mtskheta: Arkeologiuri k'vleva-dziebis shedegebi, T'omi IV. Samtavro. Ts'inaant'ik'uri khanis arkeologiuri dzeglebi (Mtskheta: Results of Archaeological Research Vol. IV. Samtavro. Archaeolgoical Monuments of the Pre-Antique Period),* Metsniereba, Tbilisi.
Kalantarian, A. A. (ed.), 2007, *Hin Hayastani oskin : m.tʿ.a. III hazaramyak-m.tʿ. XIV dar (Gold of Ancient Armenia: III millennium BC-A.D. 14th c.),* HH GAA "Gitutʿyun" hratarakchʿutʿyun, Erevan.
Kavtaradze, G. L., 1983, *K khronologii epokhi eneolita i bronzy,* Metsniereba, Tbilisi.
Kuftin, B. A., 1941, *Arkheologicheskie Raskopki v Trialeti,* Akademii Nauk Gruzinskoj SSR, Tbilisi.
Kuftin, B. A., 1949, *Arkheologicheskaya Marshrutnaya Ekspeditsiya 1945 goda v Yugo-Osetiyu i Imeretiyu (Archaeological Expedition of 1945 to South Ossetia and Imereti),* Akademii Nauk Gruzinskoy SSR, Tbilisi.
Kushnareva, K. K., 1993, *Yuzhnyy Kavkaz v IX-II Tys. do n. e. Etapy Kul'turnogo i Sotsial'no-Ekonomicheskogo Razvitiya,* RAN IIMAK, St. Petersburg.
Kushnareva, K. K., 1997, *The southern Caucasus in prehistory: stages of cultural and socioeconomic development from the eighth to the second millennium B.C,* University Museum Monograph 99*,* University of Pennsylvania Museum, Philadelphia.
Kvavadze, E. and Kakhiani, K., 2010, Palynology of the Paravani burial mound (Early Bronze Age, Georgia), *Vegetation History and Archaeobotany,* **19**, 469-478.
Lordkipanidze, N., 2016, Samk'auli ananauris №3 qorghanidan (Jewelry from Ananauri Kurgan №3), in *Ananauri Didi Q'organi №3 (Ananauri Big Kurgan №3)* (eds. Z. Makharadze, N. Kalandadze, and B. Murvanidze), Georgian National Museum, Tbilisi, 107-117.
Lordkipanidze, O., 2001, "Gandzebi" kolkhur brinjaos kulturashi (punktiis depinitsiis da kulturul-sotsiologiuri interpretatsiis tsda) ("Hoards" of the Colchian Bronze Culture (an attempt at functional definition and sociological and ethnocultural interpretation)), in *Caucasus Essays on the Archaeology of the Neolithic-Bronze Age: Dedicated to the 80th Birthday of Prof. Otar Japaridze* (Center for Archaeological Studies of the Georgian Academy of Sciences, Tbilisi, 178-194.
Lyonnet, B., Akhundov, T., Almamedov, K., Bouquet, L., Courcier, A., Jellilov, B., Huseynov, F., Loute, S., Makharadze, Z., and Reynard, S., 2008, Late Chalcolithic kurgans in Transcaucasia: The cemetery of Soyuq Bulaq (Azerbaijan), *Archäologische Mitteilungen aus Iran und Turan,* **40**, 27-44.

Maisuradze, V. G. and Inanishvili, G. V., 2006, The Shilda Sanctuary, a cult monument in Kakhetia, Republic of Georgia, *Anthropology and Archaeology of Eurasia,* **45**, 29-48.
Makharadze, Z., 2018, Rich kurgan burials of the Early Bronze Age from eastern Georgia, in *Context and Connection: Studies on the Archaeology of the ancient Near East in honour of Antonio Sagona* (eds. A. Batmaz, G. Bedianashvili, A. Michalewicz, and A. Robinson), Peeters, Leuven, 409-448.
Makharadze, Z., Kalandadze, N., and Murvanidze, B. (eds.), 2016, *Ananauri Didi Q'organi №3 (Ananauri Big Kurgan №3),* Georgian National Museum, Tbilisi.
Makharadze, Z. and Murvanidze, B., 2014, Ananauris № 3 didi q'organi (Ananauri № 3 big kurgan) (in Georgian with English summary), *Dziebani,* **22**, 50-68.
Manning, S. W., Smith, A. T., Khatchadourian, L., Badalyan, R., Lindsay, I., Greene, A., and Marshall, M., 2018, A new chronological model for the Bronze and Iron Age South Caucasus: radiocarbon results from Project ArAGATS, Armenia, *Antiquity,* **92**, 1530-1551.
Martirosyan, A. A., 1974, *Argishtikhinili,* Akademii Nauk Armyanskoy SSR, Erevan.
Menabde, M. and Davlianidze, T., 1968, *Mogil'niki Trialeti, Katalog I* Metsniereba, Tbilisi.
Mikeladze, T., Muskhelishvili, D., and Khakhutaishvili, D. A., 1984, Issledovaniya Kolkhidskoy arkheologicheskoy ekspeditsii (Research of the Colchian Archaeological Expedition), in *Polevyye arkheologicheskiye issledovaniya v 1981 godu (Archaeological field research in 1981)* (eds. O. Lordkipanidze, T. Mikeladze, and I. L. Jalagania), Metsniereba, Tbilisi, 22-25.
Mikeladze, T. K., 1985, *Kolkhetis Adrerkinis Khanis Samarovnebi (Urekisa da Nigvzianis Samarovnebi) (Early Iron Age Colchian Cemeteries (Ureki and Nigvziani Cemeteries)) (in Georgian with Russian summary),* Metsniereba, Tbilisi.
Mikeladze, T. K. and Baramidze, M. V., 1977, Kolkhskiy mogil'nik VII-VI vv. do n. e. v c. Nigvziani (Colchian cemetery of the VII-VI c. BC in the village Nigvziani), *Korotkiye Schyoty Instituta Arkheologii (Short Accounts of the Institute of Archaeology),* **151**, 33-39.
Mikeladze, T. K., Migdisova, N. P., and Papuashvili, R. I., 1985, Osnovnye itogi polevykh issledovaniy Kolkhidskoy Ekspeditsii (Basic results of the field research of the Colchian Expedition), in *Polevye arkheologicheskiye issledovaniya v 1982 godu (Archaeological field research in 1982)* (eds. O. Lordkipanidze, T. Mikeladze, and I. L. Jalagania), Metsniereba, Tbilisi, 37-40.
Mnatsakanyan, A. O., 1961, Lchashenskiye kurgany (raskopki 1956 goda), *Kratkie Soobshchenie Instituta Arkheologii,* **85**, 66-72.
Narimanishvili, G., 2010, Trialeti in the 15th and 14th Centuries BC, in *Rescue Archaeology in Georgia: The Baku-Tbilisi-Ceyhan and South Caucasian Pipelines* (ed. G. Gamkrelidze), Georgian National Museum, Tbilisi, 312-369.
Narimanov, I., 2004, Archaeological sites of the Early Bronze Age in north Azerbaijan: a gazetteer, in *A View from the Highlands: Archaeological Studies in Honour of Charles Burney* (ed. A. Sagona), Peeters, Herent, 467-473.
Needham, S. P., 1988, Selective deposition in the British Early Bronze Age, *World Archaeology,* **20**, 229-248.

Oganesian, V. E., 1992, A silver goblet from Karashamb, *Soviet Anthropology and Archaeology,* **30**, 84-102.
Palmisano, A., Lawrence, D., de Gruchy, M. W., Bevan, A., and Shennan, S., 2021, Holocene regional population dynamics and climatic trends in the Near East: A first comparison using archaeo-demographic proxies, *Quaternary Science Reviews,* **252**, 106739.
Papuashvili, R., 2011, K voprosu ob absolyutnoy khronologii mogil'nikov kolkhidy epokhi pozdney bronzy-rannego zheleza (On the question of the absolute chronology of the cemeteries of Colchis in the Late Bronze - Early Iron Age), in *Voprosy Drevney i Srednevekovoy Arkheologii Kavkaza (Questions of Ancient and Medieval Archaeology of the Caucasus)* (eds. Z. K. Albegova, M. K. Bagaev, and S. N. Korenevskiy), Uchrezhdeniye Rossiyskoy Akademii Nauk Institut Arkheologii, Grozny, Russia, 82-94.
Papuashvili, R., 2012, The Late Bronze/Early Iron Age burial grounds from Tsaishi, in *Austausch und Kulturkontakt im Südkaukasus und seinen angranzenden Regionen in der Spätbronze-/Früheisenzeit* (eds. A. Mehnert, G. Mehnert, and S. Reinhold), Beier & Beran, Langenweißbach, 65-78.
Pecqueur, L., Decaix, A., and Lyonnet, B., 2017, Un kourgane de la phase Martkopi à Mentesh Tepe (Période des Premiers Kourganes, Bronze ancien), in *The Kura Projects: New Research on the Later Prehistory of the Southern Caucasus* (eds. B. Helwing, T. Aliyev, B. Lyonnet, F. Guliyev, S. Hansen, and G. Mirtskhulava), Dietrich Reimer, Berlin, 179-192.
Piotrovskiy, B. B., 1950, *Karmir-Blur I: Rezultaty Raskopok 1939-1949,* AN Armyanskoy CCP, Yerevan.
Piotrovskiy, B. B., 1952, *Karmir-Blur II: Rezultaty Raskopok 1949-1950,* AN Armyanskoy CCP, Yerevan.
Piotrovskiy, B. B., 1955, *Karmir-Blur III: Rezultaty Raskopok 1951-1953,* AN Armyanskoy CCP, Yerevan.
Pitskhelauri, K. N., 1984, Kakhetskaya arkheologicheskaya ekspeditsiya, in *Polevye arkheologicheskiye issledovaniya v 1981 godu (Archaeological field research in 1981)* (eds. O. Lordkipanidze, T. Mikeladze, and I. L. Jalagania), Metsniereba, Tbilisi, 26-28.
Puturidze, M., 2003, Social and Economic Shifts inthe South Caucasian Middle Bronze Age, in *Archaeology in the Borderlands: Investigations in Caucasia and Beyond* (eds. A. T. Smith and K. S. Rubinson), Cotsden Institute of Archaeology, UCLA, Los Angeles, 111-127.
Ramishvili, A., 1981, Arkheologicheskie raskopki v. s. Tsagvli, in *Polevyye arkheologicheskiye issledovaniya v 1978 godu (Archaeological field research in 1978)* (eds. O. Lordkipanidze, T. Mikeladze, and I. Jalagania), Metsniereba, Tbilisi, 52-56.
Rösler, E., 1894, Archäologische Thätigkeit in Jahre 1893 Transkaukasien, *Zeitschrift für Ethnologie: Verhandlungern der Berline Gesellschaft für Anthropologie, Ethnologie, und Urgeschichte,* **26**, 213-241.
Rösler, E., 1896a, Archäologische Untersuchungen in Transkaukasien 1894, *Zeitschrift für Ethnologie: Verhandlungern der Berline Gesellschaft für Anthropologie, Ethnologie, und Urgeschichte,* **28**, 77-108.

Rösler, E., 1896b, Ausgrabungen bei Chodshali 1895, *Zeitschrift für Ethnologie: Verhandlungern der Berline Gesellschaft für Anthropologie, Ethnologie, und Urgeschichte,* **28**, 170-186.

Rösler, E., 1898, Neue Ausgrabungen und archäologische Forschungsreisen in Transkaukasien. (Mai und Juli 1897.), *Zeitschrift für Ethnologie: Verhandlungern der Berline Gesellschaft für Anthropologie, Ethnologie, und Urgeschichte,* **30**, 416-453.

Rösler, E., 1899, im Auftrage der Kaiserlich Russischen Archäologische Comission unternommenen archäologischen Forschungen in Transkaukasien in Jahre 1897, *Zeitschrift für Ethnologie: Verhandlungern der Berline Gesellschaft für Anthropologie, Ethnologie, und Urgeschichte,* **31**, 243-291.

Rubinson, K. S., 2013, Actual imports or just ideas? Investigations in Anatolia and the Caucasus, in *Cultures in Contact: From Mesopotamia to the Mediterranean in the Second Millennium BC* (eds. J. Aruz, S. R. Graff, and Y. Rakic), Metropolitan Museum of Art, New York, 12-25.

Sagona, A., 2000, Sos Höyük and the Erzurum region in late prehistory: A provisional chronology for northeast Anatolia, in *Chronologies des pays du Caucase et de l'Euphrate aux IVe–IIIe millénaires* (eds. C. Marro and A. Hauptmann), De Boccard, Paris, 329-373.

Sagona, A., 2018, *The Archaeology of the Caucasus: From the Earliest Settlements to the Iron Age,* Cambridge University Press, Cambridge.

Simonyan, H. and Manaseryan, N., 2013, Royal tombs with horse sacrifices in Nerkin Naver, Armenia (Middle Bronze Age), in *Archaeozoology of the Near East X: Proceedings of the Tenth International Symposium on the Archaeozoology of South-Western Asia and Adjacent Areas* (eds. B. De Cupere, V. Linseele, and S. Hamilton-Dyer), Peeters, Leuven, 173-208.

Spitsyn, A., 1909, Nekotorye Kavkazskie mogil'niki, *Izvestiya Imperatorskoy Arkhelogicheskoy Komissii,* **29**, 1-17.

Stöllner, T., Craddock, B., Gambaschidze, I., Gogotchuri, G., Hauptmann, A., Hornschuch, A., Klein, F., Löffler, I., Mindiashwili, G., Muwanidze, B., Senczek, S., Schaich, M., Steffens, G., Tamasashvili, K., Timberlake, S., Jansen, M., and Courcier, A., 2014, Gold in the Caucasus: New research on gold extractsion in the Kura-Araxes Culture of the 4th millennium BC and the early 3rd millennium BC, *Tagungen des Landesmuseums für Vorgeschichte Halle,* **11**, 71-110.

Tobler, W. 1993. Three presentation on geographical analysis and modeling. Santa Barbara: National Center for Geographic Information and Analysis.

Tushishvili, N., 1981, Rezul'taty rabot Arkheologicheskoy Ekspeditsii Algetskogo Ushchel'ya (Results of the work of the Algeti Gorge Archaeological Expedition), in *Polevye arkheologicheskiye issledovaniya v 1978 godu (Archaeological field research in 1978)* (eds. O. Lordkipanidze, T. Mikeladze, and I. Jalagania), Metsniereba, Tbilisi.

Wilkinson, C. K., 1965, Art of the Marlik Culture, *The Metropolitan Museum of Art Bulletin,* **24**, 101-109.

Wolf, D., Borg, G., Pernicka, E., Meliksetian, K., Kunze, R., and Bobokhyan, A., 2011, Geoarchäologische Untersuchungen der Goldvorkommen von Sotk und Fioletovo, Armenian, in *Archäologie in Armenien. Ergebnisse der Kooperationsprojekte*

2010 – Ein Vorbericht (eds. H. Meller and P. Avetisyan), Landesamt für Denkmalpflege und Archäologie Sachsen-Anhalt, Landesmuseum für Vorgeschichte, Halle, 51-68.

Yardimiciel, A., Özdemir, M. A., and Işıklı, M., 2018, A survey project on the borderlands of Turkey – Armenia – Nakhchivan – North-western Iran: Preliminary report of the 2014-2016 surveys on the middle Araxes Basin, in *Landscape Archaeology in Southern Caucasia: Finding Common Ground in Diverse Environments* (eds. W. Anderson, K. Hopper, and A. Robinson), Austrian Academy of Sciences, Vienna, 67-81.

Zhorzhikashvili, L. G. and Gogadze, E. M., 1974, *Pamyatniki Trialeti Epokhi Panney i Credney Bronzy (raskopki 1936-1940, 1947-1948 gg.),* Metsniereba, Tbilisi.
